# Newly Discovered Components of *Dendrolimus pini* Sex Pheromone

**DOI:** 10.3390/insects13111063

**Published:** 2022-11-17

**Authors:** Krzysztof J. Rudziński, Dorota Staszek, Monika Asztemborska, Lidia Sukovata, Jerzy Raczko, Marek Cieślak, Andrzej Kolk, Rafał Szmigielski

**Affiliations:** 1 Institute of Physical Chemistry, Polish Academy of Sciences, 44/52, Kasprzaka Street, 01-224 Warsaw, Poland; 2 Forest Research Institute, 3, Braci Leśnej Street, Sękocin Stary, 05-090 Raszyn, Poland

**Keywords:** pine-tree lappet moth, sex pheromone, Scots pine, gas chromatography, mass spectrometry, wind tunnel

## Abstract

**Simple Summary:**

Larvae of the pine-tree lappet moth (*Dendrolimus pini*) feed on needles of pine trees in Europe and Asia. During outbreaks, they can massively defoliate pine forests. The discovery of unknown components of *D. pini* sex pheromone opens possibilities for optimizing the lures for trapping *D. pini* males and increasing their use in insect monitoring systems.

**Abstract:**

The pine-tree lappet moth, *D. pini,* is a harmful defoliator of pine forests in Europe and Asia and a potentially invasive species in North America. The lures for trapping *D. pini* males based on two known components of its sex pheromone appeared weakly attractive to male moths. Identification of all components of the sex pheromone might allow for the development of more effective lures. The pheromone was sampled from virgin females using SPME and analyzed using gas chromatography coupled with mass spectrometry. Four new likely components ((*Z*5)-dodecenal, (*Z*5)-dodecen-1-ol, (*Z*5)-decen-1-yl acetate, (*Z*5)-tetradecen-1-yl acetate) and two known components ((*Z*5,*E*7)-dodecadienal, (*Z*5,*E*7)-dodecadien-1-ol) were identified based on comparison against authentic standards, Kováts indices and spectra libraries. The samples also contained several sesquiterpenes. Wind tunnel and field experiments showed that some blends of synthetic pheromone components alone or enriched with Scots pine essential oil (*SPEO*) were attractive to *D. pini* males. One component—(*Z*5)-decen-1-yl acetate—had a repelling effect. The presented knowledge of *D. pini* sex pheromone provides a basis for developing optimal lures for monitoring or controlling insect populations.

## 1. Introduction

The pine-tree lappet moth, *Dendrolimus pini* L. (Lepidoptera, Lasiocampidae), inhabits pine forests of Europe and Asia, from the United Kingdom to Northern China and Far-East Russia [1,2]. It is considered a potential invasive insect in North America [3]. The insect prefers Scots pine *Pinus sylvestris* L. as a host tree but has also occurred on other pine species, firs, spruces, cedars, junipers, and hemlocks [1,3,4]. It is one of the significant Scots pine defoliators in many countries, including Poland and Germany [2]. Periodic large outbreaks of *D. pini* have occurred over centuries [5]. In Poland, the outbreak in 2011–2014 was the largest one in the previous 70 years and covered about 184,000 hectares. Trees highly defoliated by *D. pini* larvae become less vital and more vulnerable to secondary pests and environmental stress like rising groundwater. The early detection of increasing populations of insects is crucial for effective outbreak prevention. The control measures are usually conducted by applying available registered insecticides.

*Dendrolimus pini* moths fly and mate mainly from mid-July to mid-August [6]. Larvae start feeding at the turn of summer and autumn in the same year. In the late autumn, usually in November, they descend to the forest litter for overwintering. Larvae resume feeding in early spring and pupate in tree crowns or on trunks at the end of June. The primary method of assessing the *D. pini* population density in Poland is the autumnal counting of overwintering larvae in forest litter [7]. The dominance structure of hibernating larval instars may depend on the outbreak phase. For instance, young instars prevailed at the outbreak’s beginning, exceeding 80% of the total larval population (Sukovata L., unpublished data). Unfortunately, the cryptic coloration makes it hard to recognize young larvae in the Scots pine forest litter. Therefore, the counters often overlook the larvae and underestimate the population densities at the initial outbreak phases. Thus, a new method for the early detection of *D. pini* at low and increasing population densities is urgently needed. A promising solution is applying sex-pheromone traps to estimate the abundance of male moths [8].

Early studies of the pheromone composition and corresponding field experiments with baited traps showed that (*Z*5,*E*7)-dodecadienal ((*Z*5,*E*7)-12:Ald) [9,10] and (*Z*5,*E*7)-dodecadien-1-ol ((*Z*5,*E*7)-12:OH) occurred in the sex pheromone of *D. pini* [11]. The catches achieved in various countries with traps baited either with (*Z*5,*E*7)-12:Ald or with mixtures of (*Z*5,*E*7)-12:Ald and (*Z*5,*E*7)-12:OH were rather low and did not exceed one male per day and trap [10,12,13,14,15]. Light traps of the Jalas type, used without the pheromones, caught up to seven males per day per trap [14]. Low catches in the pheromone traps could result from several factors, including the incomplete composition of sex-pheromone lures, which were insufficiently attractive to the insects, too low or too high loads of the pheromone in lures, and low densities of the *D. pini* populations studied.

Therefore, we aimed to (a) unveil the complete composition of the sex pheromone of *D. pini*, (b) check if its synthetic equivalent is effective in trapping males, and (c) explore the importance of each pheromone component in mate finding. The work scope ranged from rearing the insects in the laboratory and chemical analyses of emissions of calling females through wind tunnel evaluation of male response to various compounds and their blends to final field testing of selected combinations in forest stands inhabited by *D. pini*.

## 2. Materials and Methods

### 2.1. Insects 

Larvae of *D. pini* were either collected in the field or obtained from eggs laid by females in a laboratory at the Forest Research Institute (FRI). The larvae were placed on fresh Scots pine twigs in ventilated rectangular boxes 45 × 30 × 23 cm in size and fed until the pupal stage. The cut ends of the twigs were dipped in plastic cups filled with water. When necessary, the twigs were replaced with new ones.

Pupae were extracted from cocoons, sexed by visual inspection of genital regions, moved to a laboratory at the Institute of Physical Chemistry (IPC), and housed individually in 150 mL glass beakers. The beakers were closed with perforated aluminum foil covers and equipped with cylindrical mesh supports made of acid-resistant steel to aid the emerging moths. Male and female pupae were kept separately in ventilated glass terraria shaded with black cardboard and equipped with automatic LED lighting, which simulated day and night (L17:D7) with one-hour-long dawn and dusk periods. For convenience, we inverted the diel cycles of the insects so that the scotophases started at 9 a.m. When handling and sampling the insects, we used red diode light within the wavelength range of 620–630 nm, invisible to *D. pini* moths [16]. Terraria with male and female insects were stored in separate rooms at a controlled temperature (23 ± 2 °C) and relative humidity (50–70%).

### 2.2. Collection of Female Volatiles 

We collected the volatile emissions from single, calling virgin females 0 to 3 days old under the red light directly in the beakers they were kept in. Usually, the females started to call about half an hour into the scotophase and continued with short breaks until dawn. We used the Solid Phase MicroExtraction (SPME) fibers with polydimethylsiloxane (PDMS) or polydimethylsiloxane-divinylbenzene (PDMS-DVB) coating, mounted in standard holders (Supelco, Sigma Aldrich, St. Louis, MO, USA). The fibers were 1 cm long and consisted of fused silica supports and PDMS coatings 100 μm thick or PDMS-DVB coatings 65 μm thick. Before sampling, the SPME fibers were conditioned for 30 min at 250 °C in a gas chromatograph inlet with a gentle stream of helium. The holders with conditioned or sample-loaded fibers were stored separately in glass containers filled with argon. When a female extended its abdominal tip, we inserted an SPME needle into the beaker and positioned the fiber as close as possible to the tip, usually 3–5 mm away (Supplementary Materials, Figure S1). We determined that the sampling time of 15 min was sufficient to obtain good chromatograms. We also sampled a few non-calling females and males for comparison.

### 2.3. Chemical Analyses of Female Emissions 

The volatiles collected were analyzed using a Trace 1300 gas chromatograph coupled to an ITQ700 ion-trap mass spectrometer with an EI ion source (Thermo Scientific, Waltham, MA, USA). A polar ZBWAX capillary column (30 m × 0.25 mm ID, 0.25 µm film thickness, Zebron, Anaheim, CA, USA, Phenomenex, Torrance, CA, USA) was used. Helium (99.9999%, Air Liquide) with a constant flow of 1 mL/min was a carrier gas. The temperature program started at 60 °C, held for 5 min, then increased by 10 °C/min to 250 °C and stayed at 250 °C for 5 min. The temperature of the transfer line was 250 °C. Each SPME fiber was inserted directly into the inlet of the gas chromatograph, where the analytes were desorbed for 3 min in a splitless mode. The ion source and the injector operated at a constant temperature of 250 °C. The ionization energy was always 70 eV. The scanning covered the entire available range (50–650 amu). Components of female emissions were identified by their retention times and Kováts indices and by matching their mass spectra with those of authentic standards (either purchased or synthesized with the exception of unavailable α-muurolene standard). The isomers of each analyzed compound eluted with different retention times (Figure S5, Supplementary Materials). The structural elucidation of unknown emission components was also supported by applying the NIST and Wiley mass spectra libraries.

Kováts retention indices were calculated following the gradient GC/MS conditions used [17,18]. A mixture of C_7_–C_30_ straight-chain hydrocarbons was used as a reference.

### 2.4. Chemicals, Standards, and Lure Components

The following lure components and standards were purchased from Bedoukian Research (Danbury, CT, USA):
(Z5)-dodecen-1-yl acetate(Z5)-12:OAc(*Z*5)-tetradecen-1-yl acetate(*Z*5)-14:OAc;

synthesized using known synthetic procedures:
(*Z*5)-decenal(*Z*5)-10:Ald(*Z*5)-decen-1-yl acetate(*Z*5)-10:OAc(*Z*5)-dodecenal(*Z*5)-12:Ald(*Z*5)-dodecen-1-ol(*Z*5)-12:OH(*Z*5)-tetradecenal(*Z*5)-14:Ald(*Z*5)-tetradecen-1-ol(*Z*5)-14:OH

or synthesized and purified using synthetic procedures based on Stille’s reaction [19,20]:
(*Z*5,*E*7)-dodecadienal(*Z*5,*E*7)-12:Ald(*E*5,*Z*7)-dodecadienal(*E*5,*Z*7)-12:Ald(*Z*5,*Z*7)-dodecadienal(*Z*5,*Z*7)-12:Ald(*E5*,*E*7)-dodecadienal(*E*5,*E*7)-12:Ald(*Z*5,*E*7)-dodecadien-1-ol(*Z*5*,E*7)-12:OH(*E*5,*Z*7)-dodecadien-1-ol(*E*5,*Z*7)-12:OH(*Z*5,*Z*7)-dodecadien-1-ol(*Z*5,*Z*7)-12:OH(*E*5,*E*7)-dodecadien-1-ol(*E*5,*E*7)-12:OH

The synthesis of all dienic compounds was based on a previously undescribed procedure which will be patented (Polish Patent 238522) in due time. For instance, (*Z*5,*E*7)-5,7-dodecadien-1-ol (*Z*5,*E*7-12:OH) was synthesized using a coupling reaction of the corresponding (*E*5)-6-iodo-1-[(*tert*-buthyldimethylsilyl)oxy]hex-5-en (obtained earlier from hex-5-yn-1-ol) with (*Z*1)-1-(trimethyltin)hex-1-en (obtained earlier from (*Z*1)-1-iodohex-1-en) in the presence of an organopalladium catalyst. The final product was isolated from a reaction mixture containing the four isomeric products (i.e., *ZE*/*EE*/*EZ*/*ZZ* stereoisomers = 97:2:1:0), using the low-pressure argentometric chromatography with a yield of 98%. The stereoisomeric differentiation of monoenyl and dienyl compounds was carried out using NMR NOESY spectroscopy.

Sesquiterpene standards were purchased from Sigma Aldrich Poland (β-caryophyllene, α-pinene, β-pinene, 3-carene, camphene, limonene, terpinolene, and myrcene) and BOC Sciences USA (δ-cadinene and γ-cadinene). β-selinene was identified against the main component of celery (*Apium graveolens* L.) seed oil (from Herbiness, Poland), and germacrene D—against the main component of the eastern purple coneflower (*Echinacea purpurea* (L.) Moench.) oil.

The Scots pine essential oil (*SPEO*) was obtained by steam distillation, described in the next section. A mixture of C_7_–C_30_ straight-chain hydrocarbons used to determine Kováts retention indices were purchased from Sigma Aldrich (St. Louis, MO, USA). n-Hexane was used as a solvent to prepare lures (99% analytically pure, Sigma Aldrich, St. Louis, MO, USA). Mass spectra and nuclear magnetic resonance (NMR) data, which finger-printed the identity and purity of the likely components of sex pheromone, are available in the Supplementary Materials file.

### 2.5. Steam Distillation of SPEO in a Deryng Apparatus 

Fresh Scots pine needles collected at the sites inhabited by *D. pini* were put into a round-bottom flask, then filled with distilled water and connected to a Deryng apparatus. The steam distillation lasted 3 h. The essential oil collected was dried using anhydrous sodium sulfate, filtered, and analyzed by GC/MS. The efficiency of the *SPEO* distillation was 1 mL per 180 g of fresh plant material.

### 2.6. Identification of SPEO Components by GC/MS

*SPEO* was analyzed using the GC/ITQ700 ion trap mass spectrometer. The analytical procedure was the same as for the analyses of female emissions except for the injection mode. The split injection ratio was 1:100. 1 µL (~0.8–0.9 µg) of pure *SPEO* was injected in GC/MS. The mass spectra of *SPEO* components were compared to the mass spectra of the authentic standards (except unavailable α-muurolene standard) and, additionally, to the NIST and Wiley libraries. The results were also compared with the literature data on the elution order of terpene-derived compounds [21,22,23,24]. The components firmly identified in the *SPEO* samples are listed in the Supplementary Materials.

### 2.7. Preparation of Lures and Dispensers 

Stock solutions of lures of defined composition were prepared by mixing n-hexane solutions of selected components of the *D. pini* sex pheromone in glass bottles. The lures were dispensed from 1 mL polyethylene vials with push-on hinged caps and an outer diameter of 8 mm (Kartell, Italy), which appeared optimal for luring *D. pini* with mixtures of (*Z*5,*E*7)-12:Ald and (*Z*5,*E*7)-12:OH [25]. The vials were filled with various lure solutions, capped, and hung in a gentle stream of clean air at room temperature until no liquid was visible inside. Then, they were packed into alu-foil bags, thermally sealed, and stored at −22 °C in a freezer for further use. Table 1 shows the chemical compositions of the lures prepared for testing. We used the components in proportions similar to those in SPME samples, which, however, were not equivalent to the proportions in sampled emissions. For (*Z*5,*E*7)-12:Ald and (*Z*5,*E*7)-12:OH, we used the proportions published in the literature [11].

### 2.8. Wind Tunnel

The experiments were carried out in a rectangular polycarbonate wind tunnel 2.5 m long × 0.6 m wide × 0.6 m high, constructed in-house (Supplementary Materials, Figure S2). A turbine blower provided airflow up to 1 m/s in an open or closed-loop set-up. Two activated-carbon filters mounted at the inlet and outlet of the tunnel cleaned the airstream. The tunnel was shaded with white foam boards and illuminated inside with red LED light (620–630 nm). The computer software continuously recorded air temperature, relative humidity, and airflow rate during each experiment. The test insects were placed on a take-off platform located at the outlet of the tunnel. The lure dispensers were hung on the polycarbonate mesh at the air-inlet. Three black-and-white video cameras recorded the behavior of insects at a speed of 35 frames per second.

We conducted all experiments during the insects’ scotophase at room temperature (25–30 °C), relative humidity 30–40%, and airflow varied between 0.4–0.6 m/s. In most cases, the males were flying even when no bait was placed in the air stream. Interestingly, when we were preparing the traps for hanging in the forest in daylight, many moths appeared flying around.

In each experiment, we placed three males in the tunnel and offered pure airflow or a single lure in three vials hung across the tunnel inlet. If used, *SPEO* was applied in separate vials hung next to the lure vials. We recorded the insect behavior both as video films and in hand-written notes. The strongest reaction observed was attributed to one of the behavior categories *R_i_* defined in Table 2, along with arbitrary numerical weights *w_i_* promoting the active behavior of moths (see also Supplementary Materials, Figure S3). We chose the weights equal to (*i* − 1)^2^, but any ascending values, for instance, *i*, would also work. The SM includes a wmv file with a video recording illustrating the behavior category 7.

For each lure, the experiment was repeated several times (Tables S1 and S2 in the Supplementary Materials). The effectiveness factor *f* of the lure was calculated using Equation (1):

(1) $$f=\frac{\sum_{i}\left(n_{i}w_{i}\right)}{\sum_{i}n_{i}}$$

where: *n_i_* is the number of experiments in which the behavior *R_i_* was observed, and Σ*_i_n_i_* is the total number of experiments for the given lure. The lures were compared directly by their effectiveness factors.

### 2.9. Field Experiments 

Four field experiments were performed. Three experiments were set up in the 63-years-old Scots pine forest in the Krucz Forest District (52°46′ N, 16°25′ E) in July and August 2015. The fourth experiment was set up in the 73-years-old Scots pine forest in the Wronki Forest District (52°46′ N, 16°15′ E) in July 2016. The density of *D. pini* populations was estimated by counting the larvae found in the crown of the trees cut down onto a canvas (one tree per forest) in June each year. There were 17 and 13 larvae of the fifth and sixth instars per tree in 2015 and 2016, respectively.

In all experiments, the vial dispensers loaded with the tested mixtures of compounds were mounted beneath the lids of white polyethylene cross-vane traps IBL-5 supplied by the Chemipan R&D Laboratories, Poland (Supplementary Materials, Figure S4). Those traps were found to be the most effective ones for capturing *D. pini* males [25]. Square vanes (20 cm × 20 cm) made of polypropylene cellular board (Tekpol^®^) were mounted between a trap’s lid, and a funnel screwed onto a polyethylene collector. Each collector had a drainage hole with a metal mesh in the bottom. A cardboard strip (3.5 cm × 3 cm) impregnated with the insecticide (7% transfluthrin) (Bros Co., Ltd., Poznań, Poland) was added to each collector to kill the trapped moths.

All experiments were set in a randomized complete block design. The distances between the blocks and between the traps in the blocks were 20–30 m. The traps were hung on pine branches, 4–6 m above the ground, using a telescopic entomological pole that ended with a hook. The traps were checked and emptied twice in experiments 1–3 (with a rotation after the first check) and once in experiment 4.

Experiment 1 lasted seven days, from 24 to 31 July, and aimed to compare the effectiveness of two lures, *STD* and *MD10* (Table 1), with eight replicates per lure. The *STD* lure contained two pheromone components known to date ((*Z*5,*E*7)-12:Ald, (*Z*5,*E*7)-12:OH), and *MD10* was the complete lure, which included two known and three newly discovered components of the pheromone ((*Z*5)-12:Ald, (*Z*5)-12:OH, (*Z*5)-14:OAc), (*Z*5)-10:OH, and *SPEO*.

Experiment 2 was carried out from 31 July to 6 August and included the *MD10* and *MD12* lures (Table 1) to evaluate the effect of *SPEO* on the effectiveness of the complete lure. Each lure was tested in 10 replicates.

In Experiment 3, which lasted from 6 to 12 August, we compared the *MD12* lure (containing six components) with five lures (*MD14*, *MD15*, *MD16, MD17,* and *MD18*—Table 1) lacking one or two components. Each lure was tested in eight replicates.

Experiment 4 was set up on 27 July 2016 and lasted until 30 July 2016. It aimed to evaluate the effect of adding (*Z*5)-10:OAc to the MD17 lure found most effective in experiment 3. Thus, we tested the *MD17* and *MD17OAc* lures, each lure in 10 replicates.

### 2.10. Statistical Analyses 

The effectiveness of the lures tested in the wind-tunnel experiments was compared using the Kruskal–Wallis ANOVA for ranks in Sigma Plot 12.5 package (academic license, Systat Software, USA), separately for the experiments with and without *SPEO*.

Before the statistical analyses of the results from the field experiments, we pooled the data from each experiment across the time scale to calculate the total number of moths per trap during the entire exposure period. The effect of the lure type on the total moth catches was evaluated using a generalized linear mixed model with a negative binomial distribution (NB_GLMM) of the dependent variable, in which the lure type and block were used as a fixed and random covariate, respectively. A Wald *χ*^2^ test was used to test the significance of fixed variables [26]. In experiment 3, the Dunnett test was applied to compare the tested lures to the control lure, i.e., *MD12*. The goodness of fit provided by the model was estimated by residual diagnostics [27,28].

All analyses were done using the R environment [29], version 3.5.1, with rStudio [30], version 1.1.463. The following R packages were used: glmmTMB [31] for NB_GLMM, emmeans [32] for the Dunnett test, and DHARMa [33] for the model diagnostics. The significance level was α = 0.05 for all analyses.

## 3. Results

### 3.1. Identification of Female Volatiles

**Figure 1:**
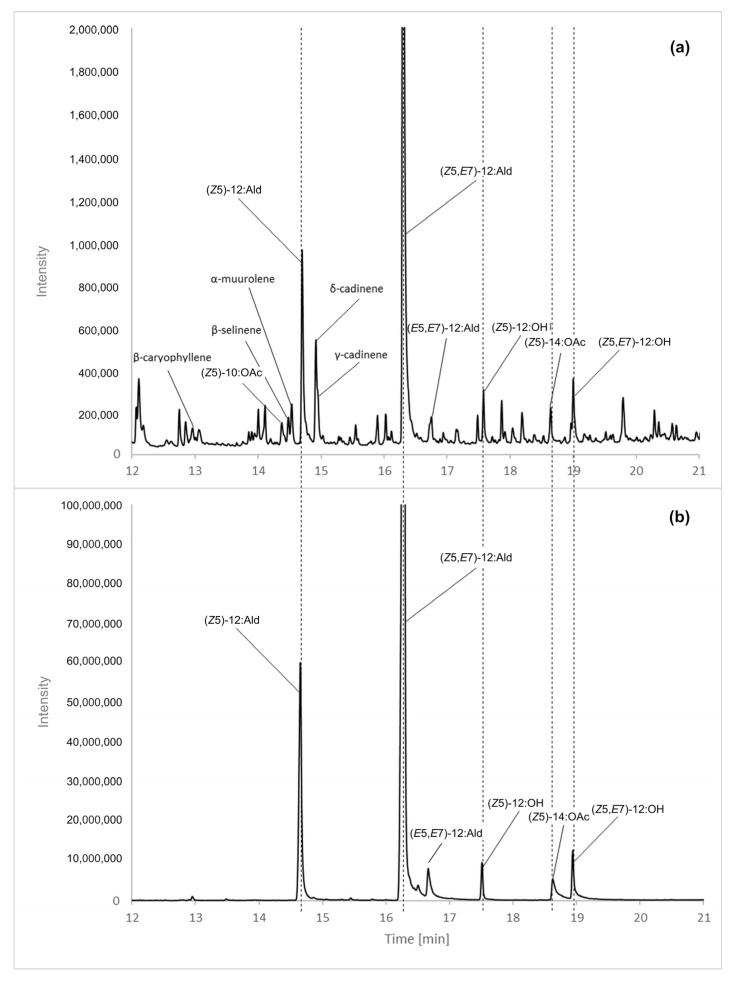
Total Ion Current (TIC) chromatograms of volatiles emitted by a calling *D. pini* female sampled with a PDMS SPME fibers (**a**), and the mixture of authentic compounds composed like *MD17* lure in Table 1 (**b**). Chromatograms were obtained using a polar ZBWAX column.

We collected and analyzed genuine samples of volatiles from calling *D. pini* females. The upper panel in Figure 1 shows a total ion current (TIC) GC/MS chromatogram of a sample collected from a one-day-old female in the 3rd h of the scotophase. The lower panel in Figure 1 shows a TIC chromatogram of a mixture of authentic compounds with a composition equivalent to the *MD17* lure (Table 1). (*E*5,*E*7)-12:Ald was included in that composition as an impurity in the isomeric (*Z*5,*E*7)-12:Ald component. For consistency, the liquid mixture was sampled using SPME and introduced to the GC/MS inlet in the same way as the pheromone samples. The peaks assigned in Figure 1 to the compounds identified are also listed in Table 3, along with their retention times, Kováts retention indices calculated from our data, and their values reported in the literature. The mass spectra of the compounds identified are shown in the Supplementary Materials. Altogether, we identified seven compounds emitted by calling *D. pini* females:

- **(*Z*5)-10:OAc** with: a diagnostic ion at *m*/*z* 138 [M-CH_3_COOH]^+**·**^, and a base peak at *m*/*z* 67 [C_5_H_7_]^+^;
- **(*Z*5)-12:Ald** with: a molecular ion at *m*/*z* 182 [M]^+**·**^, diagnostic ions at *m*/*z* 164 [M-H_2_O]^+**·**^ and *m*/*z* 138 [M-C_2_H_3_O]^+**·**^**,** and a base peak at *m*/*z* 67 [C_5_H_7_]^+^;
- **(*Z*5,*E*7)-12:Ald** with: a molecular ion at *m*/*z* 180 [M]^+**·**^, diagnostic ions at *m*/*z* 162 [M-H_2_O]^+**·**^ and *m*/*z* 137 [M-C_2_H_3_O]^+**·**^, a diagnostic ion for a 5,7-diene residue at *m*/*z* 123 [C_9_H_15_]^+**·**^ [34], and a base peak at *m*/*z* 79 [C_6_H_7_]^+^;
- **(*E*5,*E*7)-12:Ald** with: a molecular ion at *m*/*z* 180 [M]^+·^, diagnostic ions at *m*/*z* 162 [M-H_2_O]^+·^ and *m*/*z* 137 [M-C_2_H_3_O]^+·^, a diagnostic ion for the presence of a 5,7-diene residue at *m*/*z* 123 [C_9_H_15_]^+·^, and a base peak at *m*/*z* 79 [C_6_H_7_]^+^;
- **(*Z*5)-12:OH** with a base peak at *m*/*z* 67 [C_5_H_7_]^+^;
- **(*Z*5)-14:OAc** with: a hardly detectable cation *m*/*z* 255 [M+H]^+^, a diagnostic ion at *m*/*z* 194 [M-CH_3_COOH]^+**·**^ and a base peak at *m*/*z* 67 [C_5_H_7_]^+^; and
- **(*Z*5,*E*7)-12:OH** with: a weak molecular ion at *m*/*z* 182 [M]^+**·**^, a diagnostic ion at *m*/*z* 164 [M-H_2_O]^+**·**^, and a base peak at *m*/*z* 79 [C_6_H_7_]^+^ [34,35,36,37].

Furthermore, five sesquiterpenes—*β*-caryophyllene, β-selinene, α-muurolene, δ-cadinene and γ-cadinene—were identified by the *m*/*z* 204 molecular ions and characteristic patterns of sesquiterpene-specific fragment ions (*m*/*z* 189, 175, 161, 147, 133, 119, 105, 91 and 79). Comparative GC/MS analysis showed that retention times and fragmentation patterns of all compounds identified clearly matched those of purchased or synthesized standards. Thus, two alcohols, three aldehydes, and two acetates were the candidate components of *D*. *pini* sex pheromone, while sesquiterpenes were instead linked to plant material consumed by caterpillars [38,39,40,41]. We excluded the (*E*5,*E*7)-12:Ald from further experiments because its contents in the samples (assumed proportional to the peak areas) were negligible compared to the contents of its (*Z*5,*E*7)-12:Ald isomer. However, 0.3% of the (*E*5,*E*7) isomer was present in the (*Z*5,*E*7) isomer as an impurity (Figure 1, bottom panel). The proportion of those isomers was similar in SPME samples of female emissions (Figure 1, top panel). Besides, we sampled male moths and non-calling females to find that their emissions contained sesquiterpenes but not the other compounds observed in calling females (Figure S6 in Supplementary Materials).

**Table 3:** Retention times (Rt) and Kováts indices (KI) of sex-pheromone components and other volatiles found in emissions of calling *D. pini* females using GC/MS-IT and a ZBWAX column.

R_t_ min	Compound	KI Calculated	KI from Literature
12.95	β-caryophyllene	1592	1594 [42]
14.38	(*Z*5)-10:OAc	1723	no data
14.48	β-selinene	1718	1711 [42]
14.54	α-muurolene	1724	1714 [42] 1725 [43]
14.70	(*Z*5)-12:Ald	1739	1724 [44]
14.92	δ-cadinene	1760	1749 [42]
14,95	γ-cadinene	1763	1752 [42]
16.30	(*Z*5,*E*7)-12:Ald	1883	1884 [45]
16.75	(*E*5,*E*7)-12:Ald	1925	1925 [45]
17.58	(*Z*5)-12:OH	2007	2007 [44]
18.64	(*Z*5)-14:OAc	2121	2103 [44]
19.01	(*Z*5,*E*7)-12:OH	2153	2161 [45]

### 3.2. Wind-Tunnel Experiments 

**Figure 2:**
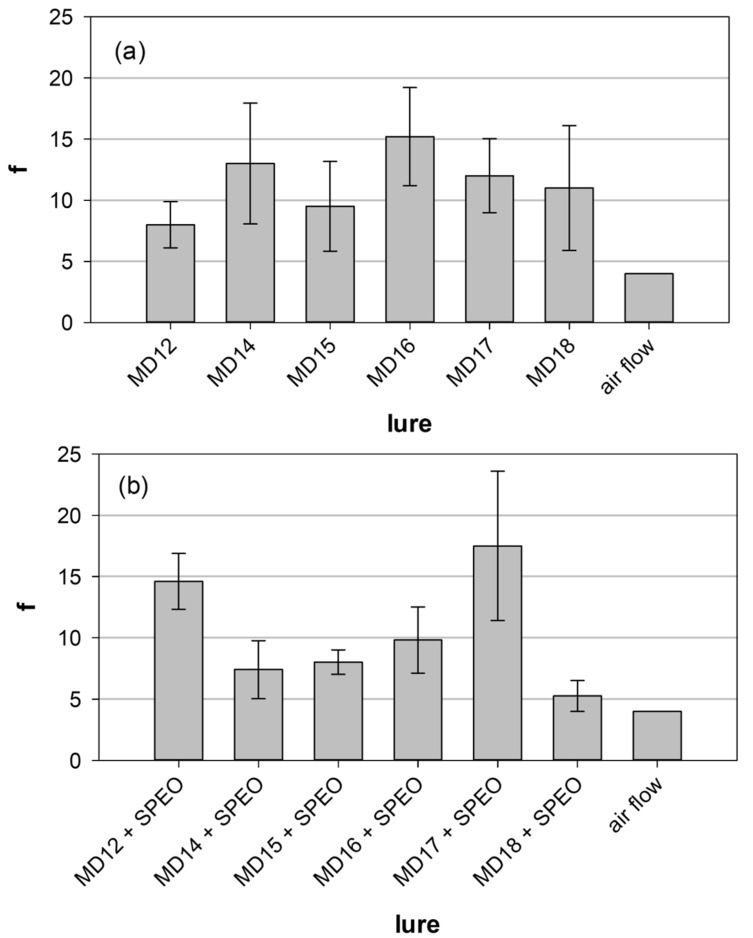
Mean effectiveness factor f of the lures tested in the wind-tunnel experiments: (**a**) lures containing the sex-pheromone components only, (**b**) lures enriched by the addition of Scots pine essential oil *SPEO* (*MD12* + *SPEO* is equivalent to *MD10*). Whiskers represent standard errors (numerical values of means and errors are given in Table S1, Supplementary Materials). The differences between the lures were not significant in all cases. For the composition of the lures, see Table 1.

The raw results of the wind tunnel experiments are shown in Tables S1 and S2 in the Supplementary Materials (lures without and with *SPEO*, respectively). Three five-component blends without *SPEO* (*MD14*, *MD16,* and *MD17*) and a four-component blend (*MD18*) appeared to be attractive to *D. pini* males (Figure 2a), but the differences between all blends were not statistically significant (Kruskal-Wallis test: *H* = 4.726, *df* = 5, *p* = 0.450). (*Z*5)-10:OH was a plausible guess-addition as a homolog of (*Z*5)-12:OH and a component of the sex pheromone of many moths like larch casebearer, *Coleoptera laricella* [46,47], turnip moth *Agrotis segetum* [48,49], and millet stem borer *Coniesta ignefusalis* [50]. The flow of pure air through the tunnel made the insects fly almost in all experiments and could serve as a reference for the lures tested.

The addition of the essential oil distilled from the Scots pine needles (*SPEO*) had no significant effect on the effectiveness of the lures in the wind tunnel tests (Figure 2) since the differences observed were not statistically significant (*H* = 3.906, *df* = 5, *p* = 0.563). Interestingly, the presence of *SPEO* in the lures slightly changed the pattern in male responses. The effectiveness of *MD17* and *MD12* lures with *SPEO* were the highest of all lures tested.

### 3.3. Field Experiments 

**Figure 3:**
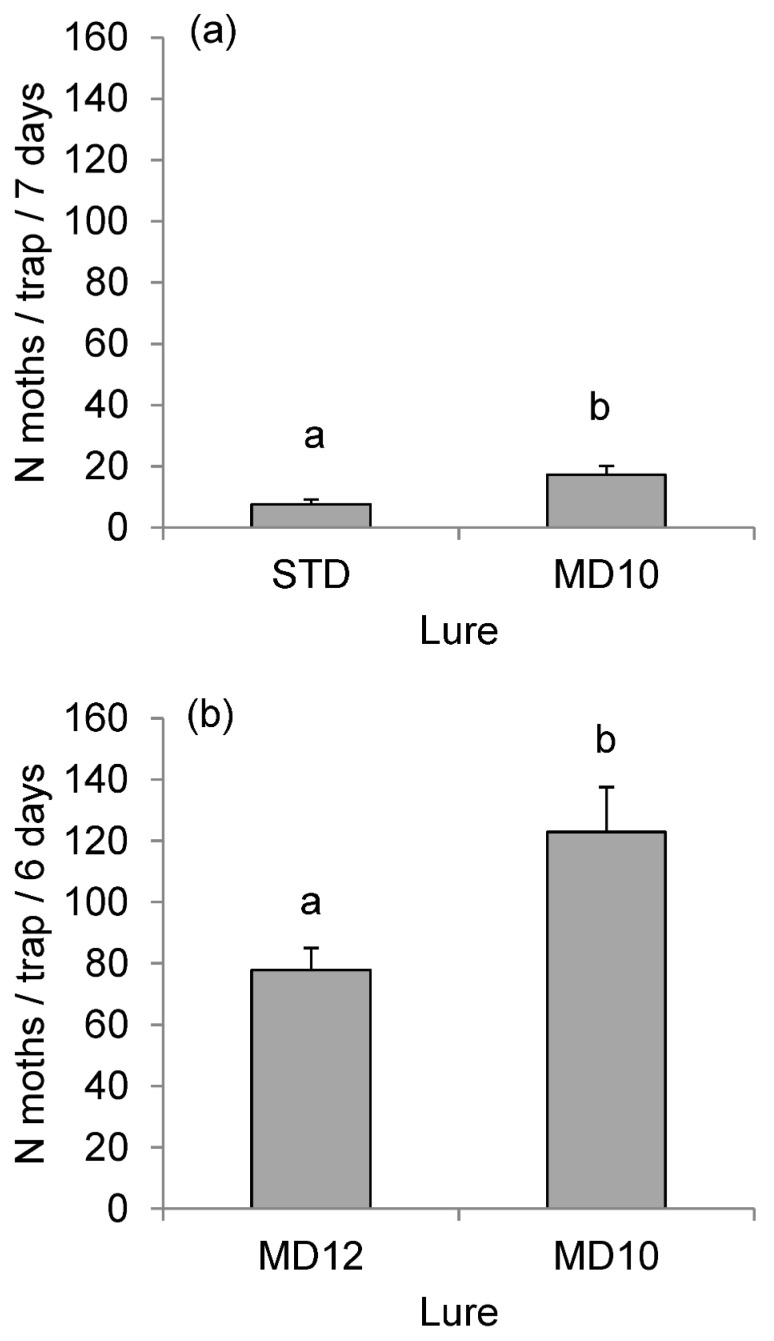
Mean (±SE) total catches per trap of *D. pini* male moths in cross-vane traps IBL-5 with different lures in (**a**) Experiment 1 from 24 to 31 July 2015 and (**b**) Experiment 2 from 31 July to 6 August 2015. The composition of the lures is explained in Table 1. Bars marked with different letters are significantly different at α = 0.05.

In Experiment 1, the *STD* and *MD10* lures differed significantly in the attractiveness to *D. pini* males (Wald test: *χ*^2^ = 8.68, *df* = 13, *p* =0.0032). During seven days of the experiment, the traps with the *MD10* lure containing six components and *SPEO* caught 17.3 ± 2.8 moths/trap, while the STD lure attracted 7.6 ± 1.5 moths/trap (Figure 3a).

Experiment 2 showed that *SPEO* had a strong significant effect on catches of *D. pini* (after excluding one outlier, *χ*^2^ = 8.95, *df* = 1, *p* = 0.0028). The traps with the *MD12* lure (without *SPEO*) caught significantly fewer males (77.8 ± 7.2 moths/trap) during the six-day exposure than the traps with the *MD10* lure, including *SPEO* (122.9 ± 14.6 moths/trap) (Figure 3b).

**Figure 4:**
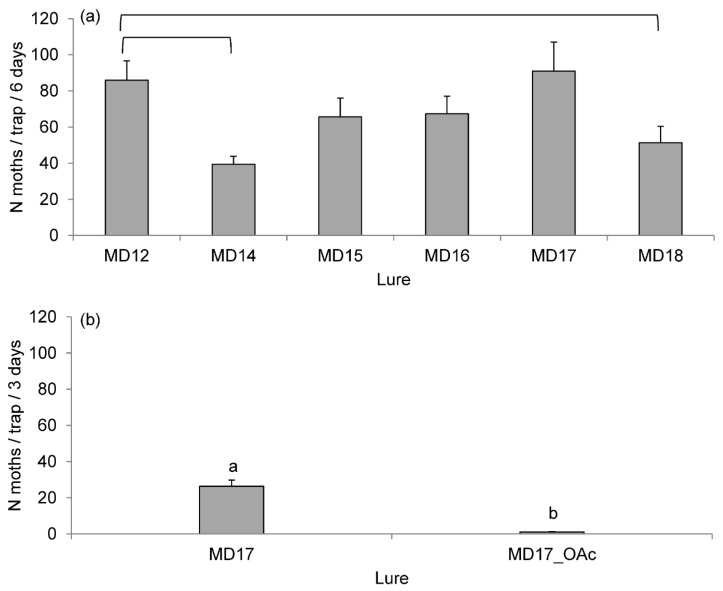
Mean (±SE) total catches per trap of *D. pini* male moths in cross-vane traps IBL-5 with different lures in (**a**) Experiment 3 from 6 to 12 August 2015 and (**b**) Experiment 4 from 27 to 30 July 2016. The composition of the lures is explained in Table 1. In panel (**a**), bars linked with horizontal brackets represent the lures significantly different from the *MD12* lure. In panel (**b**), bars marked with different letters are significantly different at *α* = 0.05.

Experiment 3 showed that removing one or two components from the six-component lure *MD12* significantly affected the moth catches in the traps (*χ*^2^ = 24.85, *df* = 5, *p* = 0.0001). During six days of the experiment, the traps with *MD17* and *MD12* lures captured the highest numbers of *D. pini* moths: 91.0 ± 16.1 moths/trap and 86.0 ± 10.6 moths/trap, respectively (Figure 4a). *MD14* lure (without (*Z*5)-12:Ald) and *MD18* lure (without (*Z*5)-12:Ald and (*Z*5)-12:OH) were the least attractive to *D. pini* males (39.4 ± 4.5 moths/trap and 51.2 ± 9.1 moths/trap, respectively). In both cases, the catches were significantly lower than in the traps with *MD12* (*MD14* vs. *MD12*: *t* = −3.85, *p* = 0.0020; *MD18* vs. *MD12*: *t* = −2.85, *p* = 0.0301). The experiment showed that the (*Z*5)-10:OH component had not improved the lures (Figure 4a, *MD12* vs. *MD17*).

In experiment 4, the addition of (*Z*5)-10:OAc to the *MD17* lure significantly reduced its attractiveness to *D. pini* males (*χ*^2^ = 85.27, *df* = 1, *p* < 0.0001). During three days, the traps with *MD17OAc* lure captured 1.0 ± 0.3 moths/trap, whereas the traps with *MD17* lure—were 26.4 ± 3.4 moths/trap (Figure 4b).

## 4. Discussion

The compounds identified as likely components of the *D. pini* sex pheromone, except (*E*5,*E*7)-12:Ald, (*Z*5)-10:OAc, and (*Z*5)-14:OAc, were observed in other *Dendrolimus* species either as the major or trace components of sex pheromones (Table 4). (*Z*5,*E*7)-12:OH was the only component common for all *Dendrolimus* species. (*Z*5)-14:OAc occurred in the sex pheromone of several moths: the Cossidae family [51], heart and dart moth *Scotia exclamationis* L. (Lepidoptera, Noctuidae) [52], and army cutworm moth *Euxoa auxiliaris* (Grote) (Lepidoptera, Noctuidae) [53]. (*Z*5)-10:OAc was the major component of the sex pheromone of the turnip moth *Agrotis segetum* (Den. et Schiff.) [54,55,56], and black cutworm moth *Agrotis ipsilon* (Hufnage) [57,58] (Lepidoptera, Noctuidae). In our studies, (*Z*5)-14:OAc had no significant effect on the attraction of *D. pini* male moths—the lure from which it was dropped out (*MD16*) became only slightly less attractive than *MD12*. In contrast, (*Z*5)-10:OAc had a repelling effect because it reduced the catches of moths when added to *MD12* (Figure 4b). In a similar experiment, (*Z*5)-10:OAc reduced the attracting power of a (*Z*5)-10:OH-based lure towards males of the larch casebearer moth, *Coleophora laricella* (Hübner) (Lepidoptera, Coleophoridae) [46,47,59]. The roles tentatively assigned to (*Z*5)-10:OAc included species isolation and attraction masking by mated female moths. Components reducing the attractiveness of sex pheromones can also play other roles, e.g., (*Z*11)-16:OH in the cotton bollworm moth *Helicoverpa armigera* (Hübner) (Lepidoptera, Noctuidae) allowed male moths recognize the sexual maturity of females [60]. Yet another acetate, i.e., (*Z*5,*E*7)-12:OAc, reduced the field catches of *Dendrolimus superans sibiricus* Tschetv [61]. Experiments 1 and 2 showed indirectly that the *MD12* lure was more attractive than the *STD* lure (Figure 3). Experiment 3 indicated that (*Z*5)-12:Ald was the only significantly effective new component of the *D. pini* lures for intraspecific communication, i.e., mate-finding. The lures without it (*MD14* and *MD18*) were significantly less effective. Other new components appeared unimportant for attracting conspecific males.

Judging by the respective peak areas, (*E*5,*E*7)-12:Ald, the geometrical isomer of (*Z*5,*E*7)-12:Ald, was detected in very small quantities. We had not added it to the lures tested in the laboratory and field experiments, but it always accompanied (*Z*5,*E*7)-12:Ald as the impurity at a level similar to that observed in female emission samples. Priesner et al. [10] found that the addition of another isomer (*E*5,*Z*7)-12:Ald or the acetate (*Z*5,*E*7)-12:OAc effectively disrupted the captures of *D. pini* males in traps baited with (*Z*5,*E*7)-12:Ald. They suggested that such inhibitory action could isolate *D. pini* from other sympatric *Dendrolimus* species. On the other hand, Ando et al. [74] found the (*Z*5,*E*7)-12:OH and (*E*5,*E*7)-12:OH isomers in extracts of abdominal tips of *Dendrolimus spectabilis* (Butler). They showed that only (*Z*5,*E*7)-12:OH out of the four possible synthetic isomers (*Z*5,*Z*7), (*Z*5, *E*7), (*E*5,*Z*7) and (*E*5,*E*7) successfully attracted male moths to the traps in field experiments.

The total catches of *D. pini* male moths in the traps with our best lures *MD10*, *MD12,* and *MD17* ranged from 17.3 insects in 7 days to 122.9 insects in 6 days, i.e., 2.5 to 20.5 insects per trap and day. Daily catches per trap estimated from the total catches reported in the literature were lower than one insect in all cases [10,12,13,14,15]. These authors used the pheromone traps of various types baited with (*Z*5,*E*7)-12:Ald alone or combined with (*Z*5,*E*7)-12:OH. None of them used the cross-vane traps or polyethylene vial dispensers shown to be the most effective [25] and used in this work. Moreover, they did not provide data on the *D. pini* population densities. Therefore, it is rather difficult to compare their results with ours directly.

Host plant volatiles can play additive or synergistic roles in pheromone communication [75,76]. They can influence pheromone production and liberation in female insects and increase the catches of males when added to the lures. We identified several sesquiterpenes emitted by *D. pini* females (Table 3 and Supplementary Materials). These compounds might originate from pine trees on which the caterpillars had fed [40] and potentially could play some role in attracting male moths. Therefore, we examined *SPEO,* a mixture of volatiles emitted by the Scots pine—a host tree of *D. pini*. In the wind tunnel experiment, it did not significantly affect the response of *D. pini* males to the lures but slightly changed its pattern (Figure 2). The field test clearly showed that *SPEO* did play some important role in mate-finding because it significantly increased the effectiveness of *MD12* (Figure 3b). The role *SPEO* played in the tunnel could be quite different from that in the field test. One may speculate that *SPEO* was just a “replacement” for missing host trees in the tunnel.

In addition to the main observations, we also confirmed that the red LED light (wavelength range 620–630 nm) does not distract *D. pini* in the scotophase period, which significantly facilitates the work with these insects in a laboratory framework.

In summary, we identified (*Z*5)-12:Ald, (*Z*5)-12:OH, (*Z*5)-10:OAc, and (*Z*5)-14:OAc as new likely components of the sex pheromone emitted by *D. pini* female moths, which accompanied the previously discovered components (*Z*5,*E*7)-12:Ald and (*Z*5,*E*7)-12:OH. Besides, we identified trace amounts of (*E*5,*E*7)-12:Ald, which may play an important role in insect communication, particularly in distinguishing species of the same genus inhabiting the same environment; thus, it is worth further research. We proved that (*Z*5)-12:Ald significantly attracted the male moths while (*Z*5)-10:OAc had a repelling effect. Thus, further work is warranted to unveil the exact roles of other discovered compounds. The mixture of all components identified but (*Z*5)-10:OAc, which has a repellent effect, is an effective lure for *D. pini* males and provides a base for further work to optimize the lure composition for use in the trap-based monitoring of *D. pini* populations. For instance, the lures lacking some components may be sufficiently effective while easier and cheaper to prepare. Our results indicate that the optimization may start from the *MD17* lure lacking (*Z*5)-10:OH. The lure should be applied either in cross-vane traps or Unitraps with polyethylene vial dispensers [25,77]. The addition of *SPEO* enhances the performance of the lure. Thus, further work is required to reveal its composition across Scots pine chemotypes and understand its role in *D*. *pini* communication.

## Figures and Tables

**Table 1:** Compositions of lures used in the laboratory and in field experiments ^a^.

Components	Lure Composition (mg per Dispenser)								
	*STD* ^b^	*MD10*	*MD12*	*MD14*	*MD15*	*MD16*	*MD17*	*MD18*	*MD17OAc*
(*Z*5,*E*7)-12:Ald	0.6	0.6	0.6	0.6	0.6	0.6	0.6	0.6	0.6
(*Z*5,*E*7)-12:OH	0.4	0.4	0.4	0.4	0.4	0.4	0.4	0.4	0.4
(*Z*5)-12:Ald	-	0.15	0.15	-	0.15	0.15	0.15	-	0.15
(*Z*5)-12:OH	-	0.15	0.15	0.15	-	0.15	0.15	-	0.15
(*Z*5)-10:OH	-	0.05	0.05	0.05	0.05	0.05	-	0.05	-
(*Z*5)-14:OAc	-	0.15	0.15	0.15	0.15	-	0.15	0.15	0.15
(*Z*5)-10:OAc	-	-	-	-	-	-	-	-	0.15
*SPEO*	-	1	-	-	-	-	-	-	-
Field Expt 1									
Field Expt 2									
Field Expt 3									
Field Expt 4									

^a^ Last row shows the sets of lures compared in four field experiments (Expt) described in the corresponding subsection; ^b^ Proportion of (*Z*5,*E*7)-12:Ald and (*Z*5,*E*7)-12:OH was adopted from Kovalev et al. [11].

**Table 2:** Categories of insect behavior in the wind tunnel experiments.

Category Index *i*	Behavior Category *R_i_*	Category Weight, *w_i_*
1	No reaction	0
2	Wing-fanning	1
3	Flight initiation	4
4	Approaching the lure, departure	9
5	Landing close to the lure, departures, returns	16
6	Landing close to the lure and staying there for a long time (>10 s)	25
7	Landing directly on the lure, attempt to copulate	36

**Table 4:** Components of sex pheromone in females of various *Dendrolimus* species (p—likely components identified in this work; r—components reported in the literature).

Compound	*D. pini*	*D. houi*	*D. kikuchii*	*D. punctatus*	*D. spectabilis*	*D. superans*	*D. superans sibiricus*	*D. tabulaeformis*
(*Z*5)-10:OAc	p							
(*Z*5)-12:Ald	p					r [62,63]		
(*Z*5)-12:OH	p	r [45] ^c^		r [64] ^c^		r [62,63]		r [65,66]
(*Z*5)-12:OAc			r [67]	r [64] ^c^				r [65,66]
(*Z*5,*E*7)-12:Ald	r [10,11], p				r [62,68]	r [62,63]	r [61] ^ac^, r [69]	
(*E*5,*Z*7)-12:Ald		r [45,70] ^c^						
(*E*5,*E*7)-12:Ald	p ^b^							
(*Z*5,*E*7)-12:OH	r [11], p	r [45] ^c^	r [62,67,71] ^c^	r [64,72,73]	r [37,62,68,74]	r [62,63]	r [61] ^ac^, r [69]	r [62,65,66]
(*E*5,*Z*7)-12:OH		r [45,70]						
(*E*5,*E*7)-12:OH		r [45] ^c^			r [37] ^c^, r [74]			
(*Z*5,*E*7)-12:OAc		r [45] ^c^	r [62,67,71]	r [64,72,73]	r [62,68]		r [69] ^c^	r [62,65,66]
(*E*5,*Z*7)-12:OAc		r [45,70]						
(*Z*5,*Z*7)-12:OAc		r [45] ^c^						
(*E*5,*E*7)-12:OAc		r [45] ^c^						
(*Z*5,*E*7)-12:OPr				r [64,72,73]	r [68] ^c^			r [62,65]
(*Z*5)-14:OAc	p							

^a^—tentatively identified and confirmed in field tests; ^b^—assumed quantitatively negligible and excluded from the experiments; ^c^—trace component.

## Data Availability

Raw data from wind-tunnel experiments are given in Supplementary Materials; field data are available on request from Lidia Sukovata.

## Supplementary Materials

The following supporting information can be downloaded at: https://www.mdpi.com/article/10.3390/insects13111063/s1, Figure S1: Sampling of volatile emission from *D. pini* females; Figure S2: Wind tunnel; Figure S3: The behavior of *D. pini* males in the wind tunnel; Figure S4: The IBL-5 trap used in the field experiments; 70eV EI ION TRAP MASS spectra of compounds identified in this work; NMR spectra of likely components od *D. pini* sex pheromone; List of compounds identified in Scots pine essential oil; Figure S5: Chromatographic separation of isomers of compounds in *D. pini* emissions; Figure S6: Comparison of emissions from a male and a non-calling female; Table S1: Tunnel experiments without *SPEO*—raw observations; and Table S2: Tunnel experiments with *SPEO*—raw observations), and a video file *behavior_7.wmv* containing a recording of the utmost behavior of males in the wind tunnel. Reference [78] is cited in the supplementary materials.
