# Supplementary material for: Newly Discovered Components of Dendrolimus pini Sex Pheromone
Source: Insects. 2022 Nov 17;13(11):1063. doi: 10.3390/insects13111063 (PMC9699269; doi:10.3390/insects13111063)
Supplement: Supplementary file 1 [file insects-13-01063-s001.zip › SubMat_ proof.pdf]

Supplementary Materials

INSECTS

Newly discovered components of *Dendrolimus pini* sex pheromone

Krzysztof J. Rudziński[a*], Dorota Staszek[a†‡], Monika Asztemborska[a], Lidia Sukovata[b], Jerzy Raczko[a§], Marek Cieślak[a], Andrzej Kolk[b,c], Rafał Szmigielski[a*]

[a]*Institute of Physical Chemistry, Polish Academy of Sciences, 44/52, Kasprzaka Street, 01-224 Warsaw, Poland*
[b]*Forest Research Institute, 3, Braci Leśnej Street, Sękocin Stary, 05-090 Raszyn, Poland*
[c] professor Andrzej Kolk passed away on 6th October 2020

Contents

Figure S1. Sampling of volatile emission from *D. pini* females.. p. 2
Figure S2. Wind tunnel .... p. 3
Figure S3. The behavior of *D. pini* males in the wind tunnel .... p. 3
Figure S4. The IBL-5 trap used in the field experiments .... p. 4
70eV EI ION TRAP MASS spectra of compounds identified in this work .... p. 5
(*Z*5,*E*7)-12:OH, (*Z*5,*E*7)-12:Ald .... p. 5
(*E*5,*E*7)-12:Ald, (*Z*5)-12:OH .... p. 6
(*Z*5)-12:Ald, (*Z*5)-10:OAc .... p. 7
(*Z*5)-14:OAc, β-caryophyllene .... p. 8
δ-cadinene, γ-cadinene .... p. 9
β-selinene, α-muurolene .... p. 10
Figure S5. Chromatographic separation of isomers of compounds in *D. pini* emissions .... p. 12
NMR spectra of likely components od *D. pini* sex pheromone.. p. 13
List of compounds identified in Scots pine essential oil .... p. 13
Figure S6. Comparison of emissions from a male and a non-calling female .... p. 14
Table S1. Tunnel experiments without *SPEO* – raw observations .... p. 15
Table S2. Tunnel experiments with *SPEO* – raw observations .... p. 15
References .... p. 15

[*] Correspondence to: Krzysztof J. Rudziński or Rafał Szmigielski, *Institute of Physical Chemistry, Polish Academy of Sciences, 44/52, Kasprzaka Street, 01-224 Warsaw, Poland*. E-mail: kjrudz@ichf.edu.pl, E-mail: ralf@ichf.edu.pl.
[†] Krzysztof J. Rudziński and Dorota Staszek should be considered joint first author
[‡] present address: *Institute of Organic Chemistry, Polish Academy of Sciences, 44/52, Kasprzaka Street, 01-224 Warsaw, Poland*
[§] present address: raczkojerzy@gmail.com

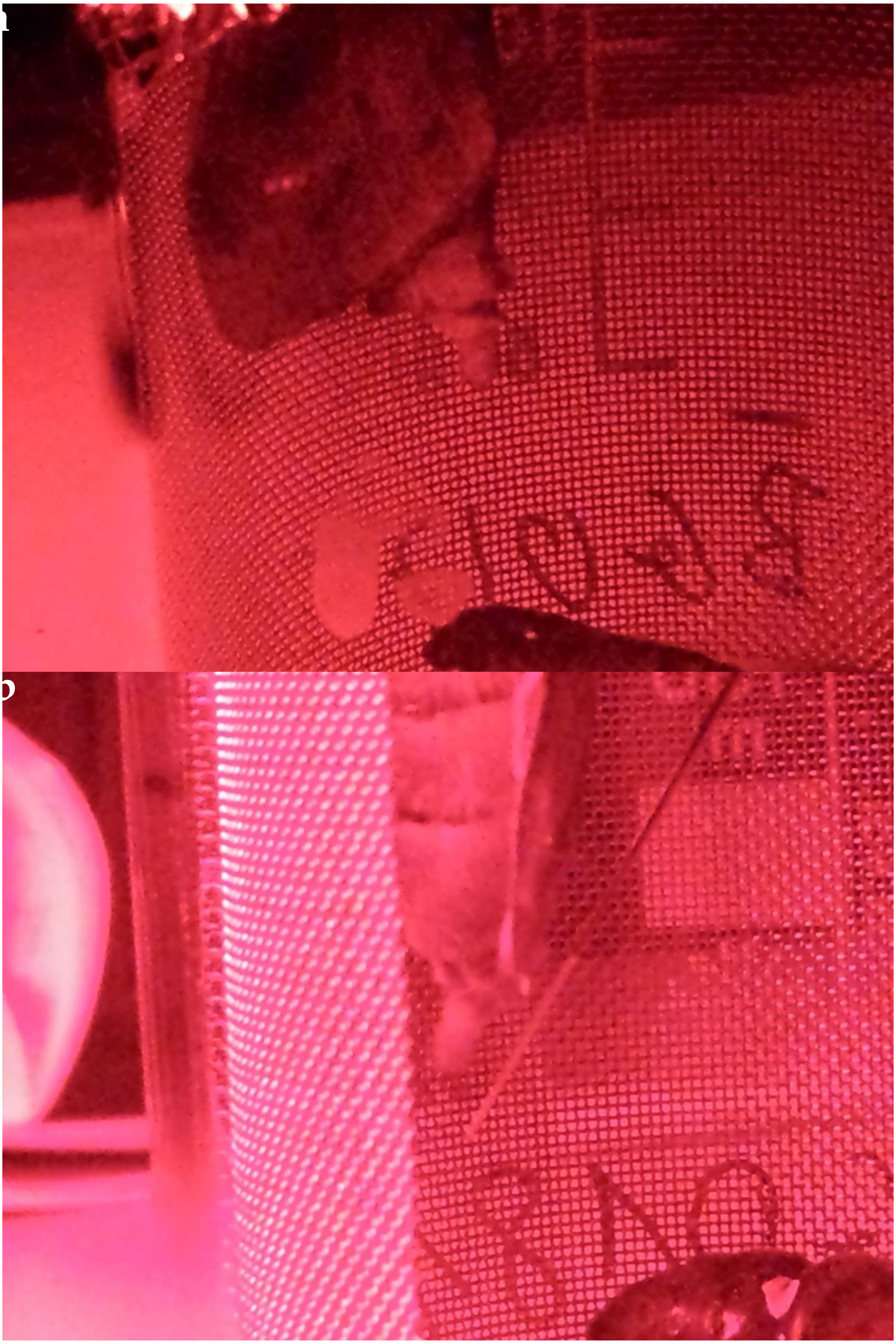

Figure S1. Sampling of volatile emission from *D. pini* females: (a) a calling female (b) placement of an SPME adsorbent fiber.

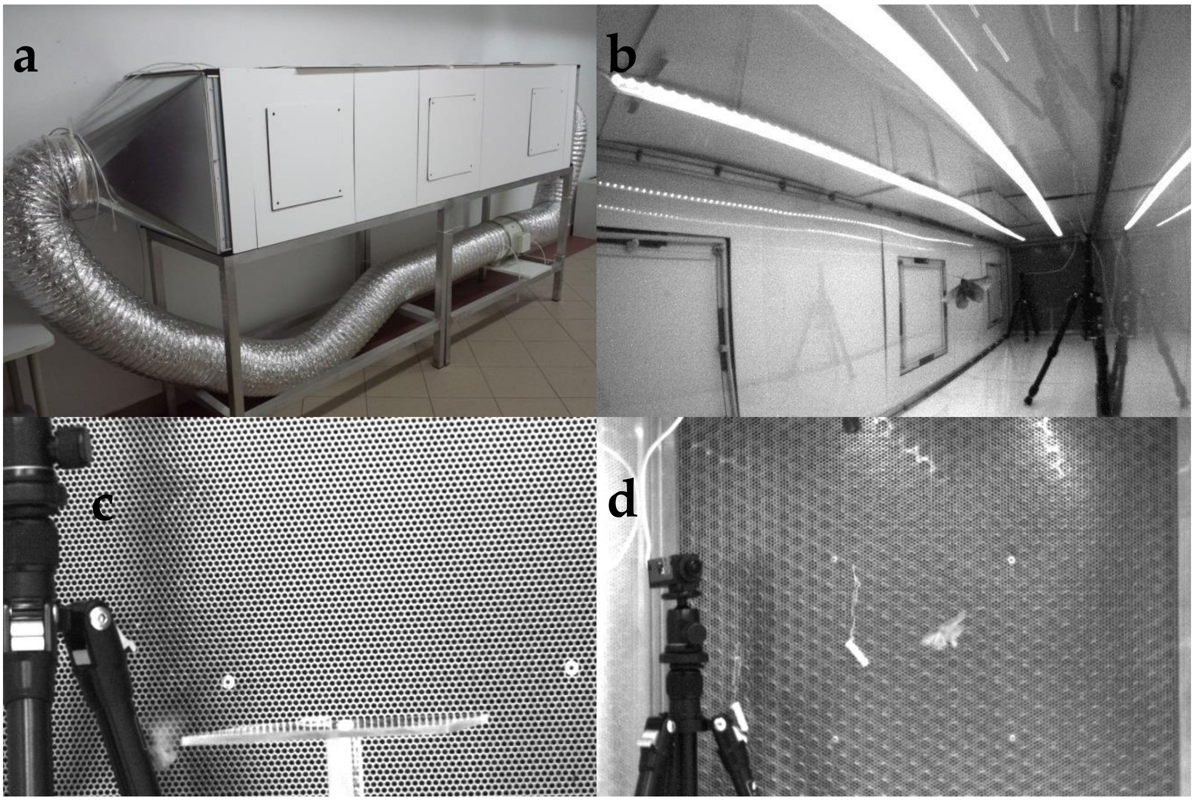

Figure S2. Wind tunnel: (a) general view; (b) inner view towards a starting platform, in the center – a flying moth; (c) starting platform; (d) air inlet, in the center – a bait vial and approaching moth.

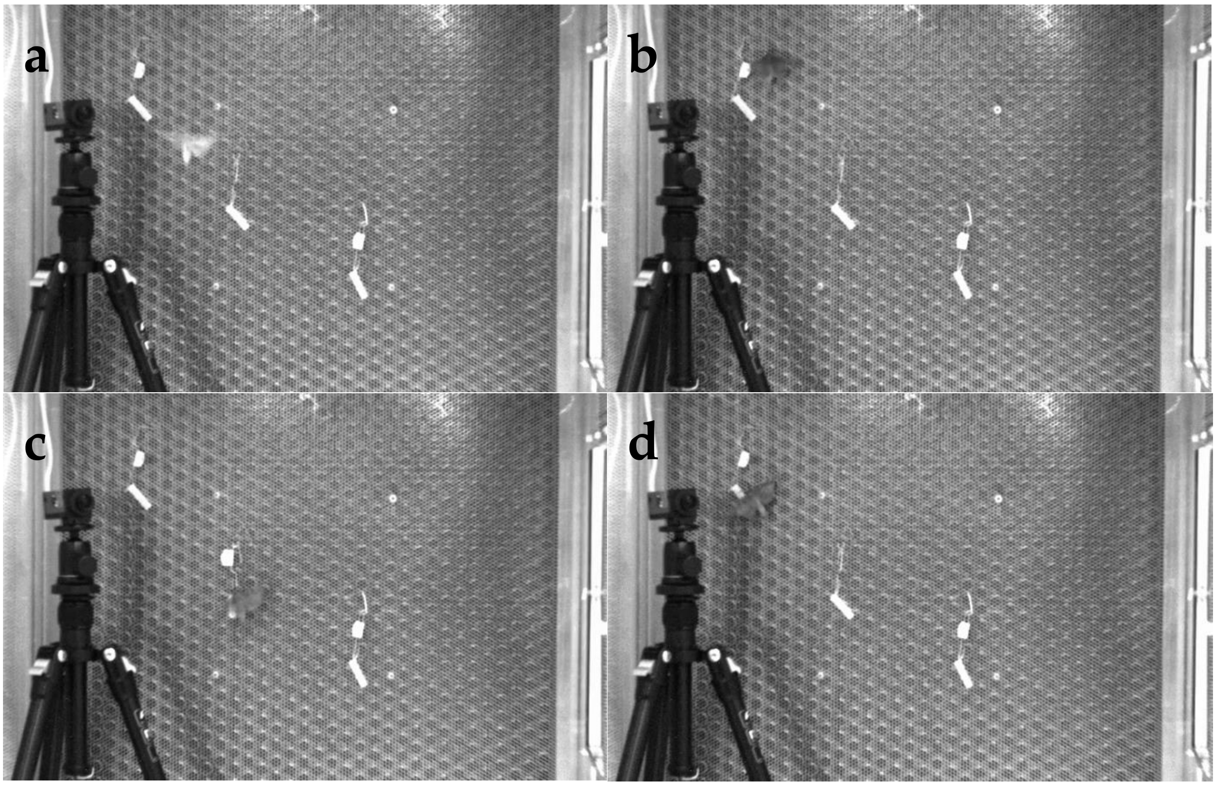

Figure S3. The behavior of *D. pini* males in the wind tunnel: (a) Approaching the lure, departing; (b) Landing close to the lure and staying there for a long time; (c,d) Landing directly on the lure, attempt to copulate.

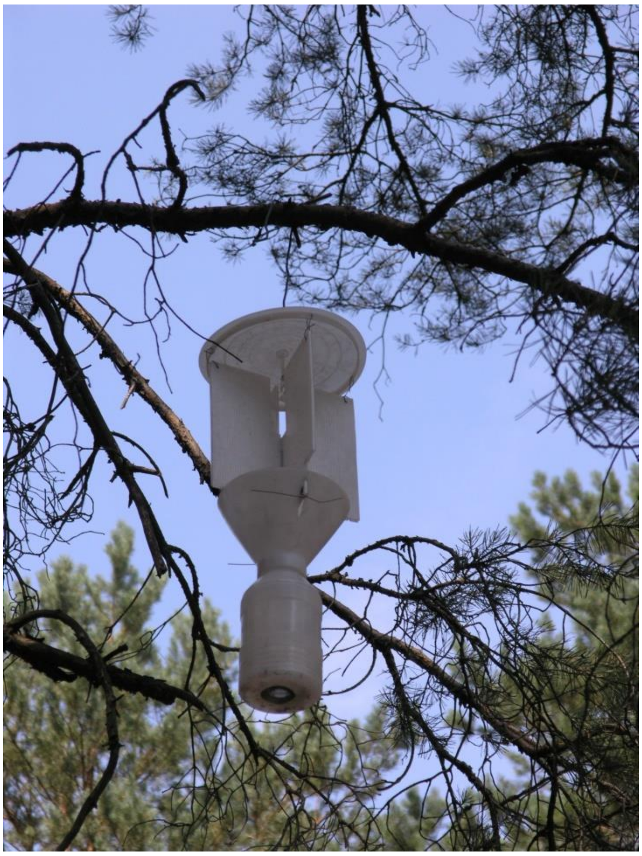

Figure S4. The IBL-5 trap used in the field experiments.

70Ev EI ION TRAP MASS SPECTRA OF SEX-PHEROMONE COMPONENTS IDENTIFIED

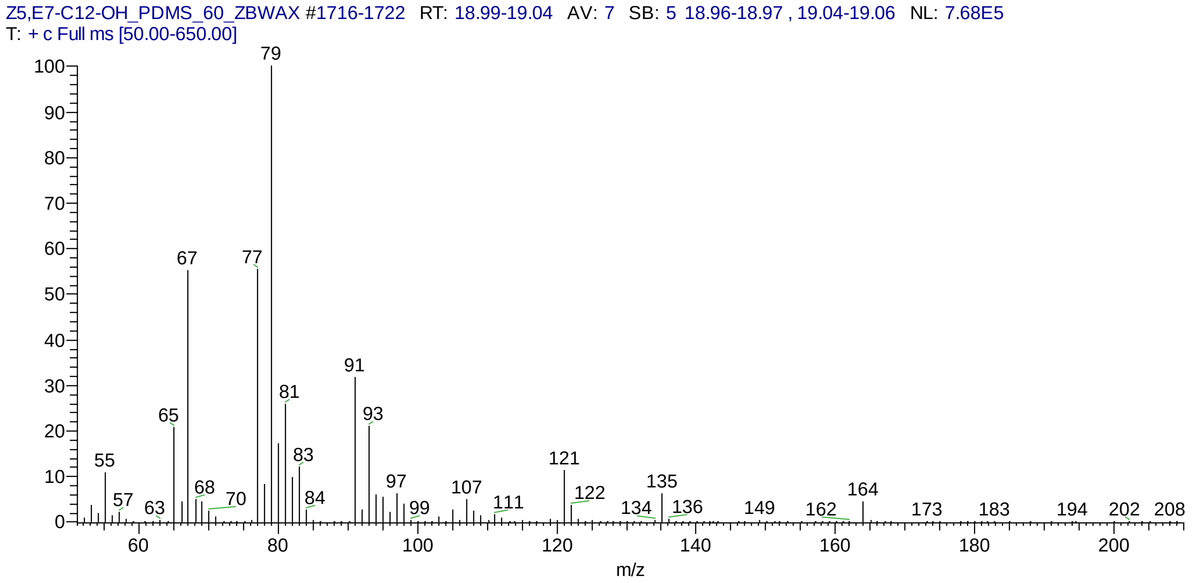

(*Z*5,*E*7)-12:OH

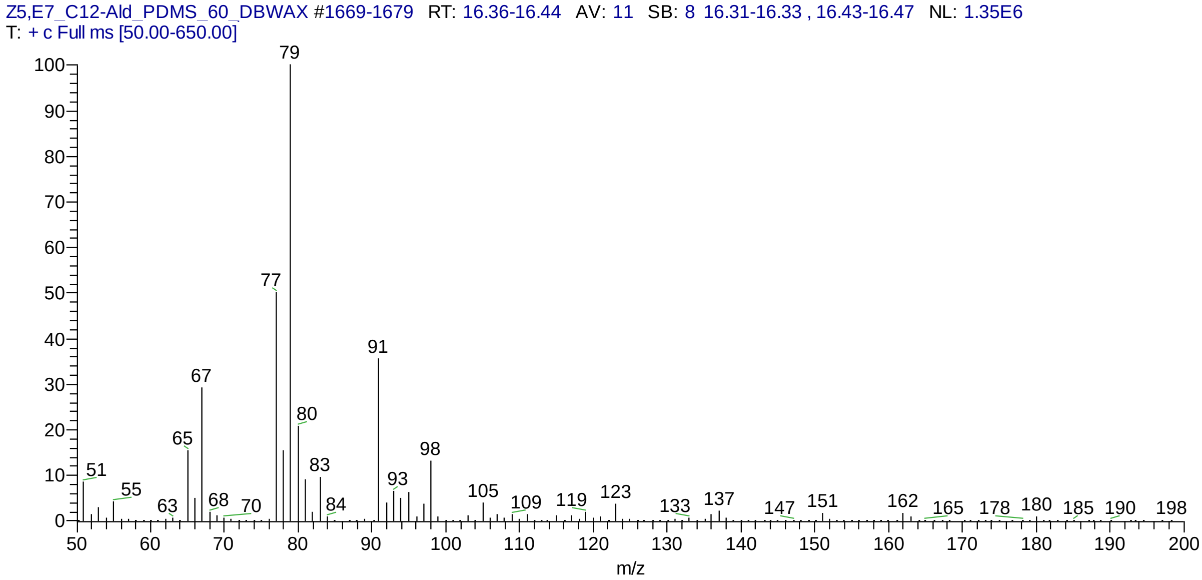

(*Z*5,*E*7)-12:Ald

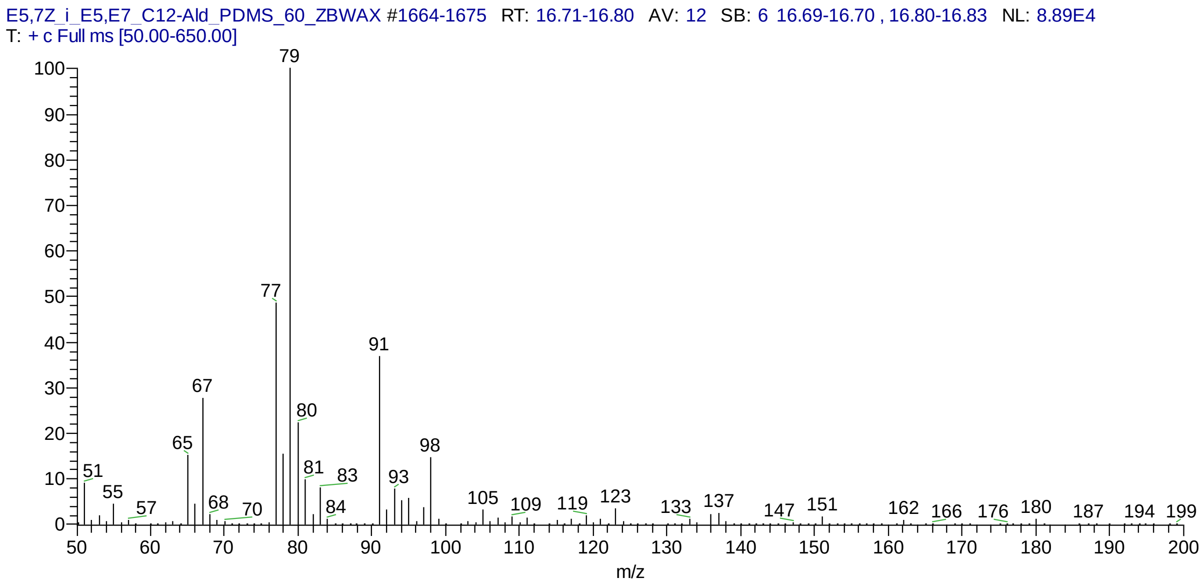

(*E*5,*E*7)-12:Ald

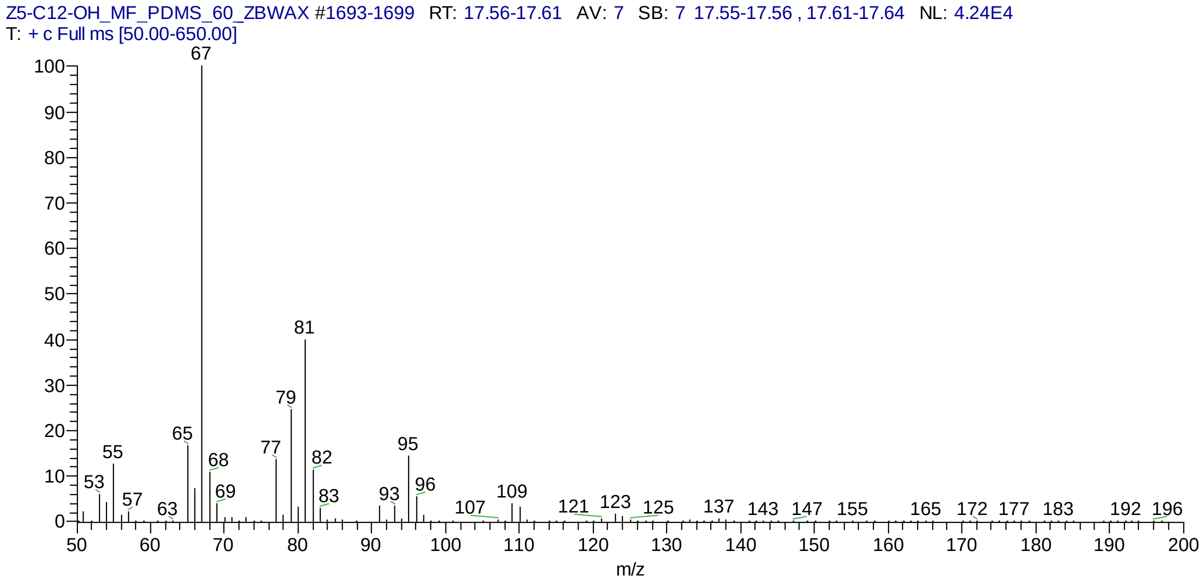

(*Z*5)-12:OH

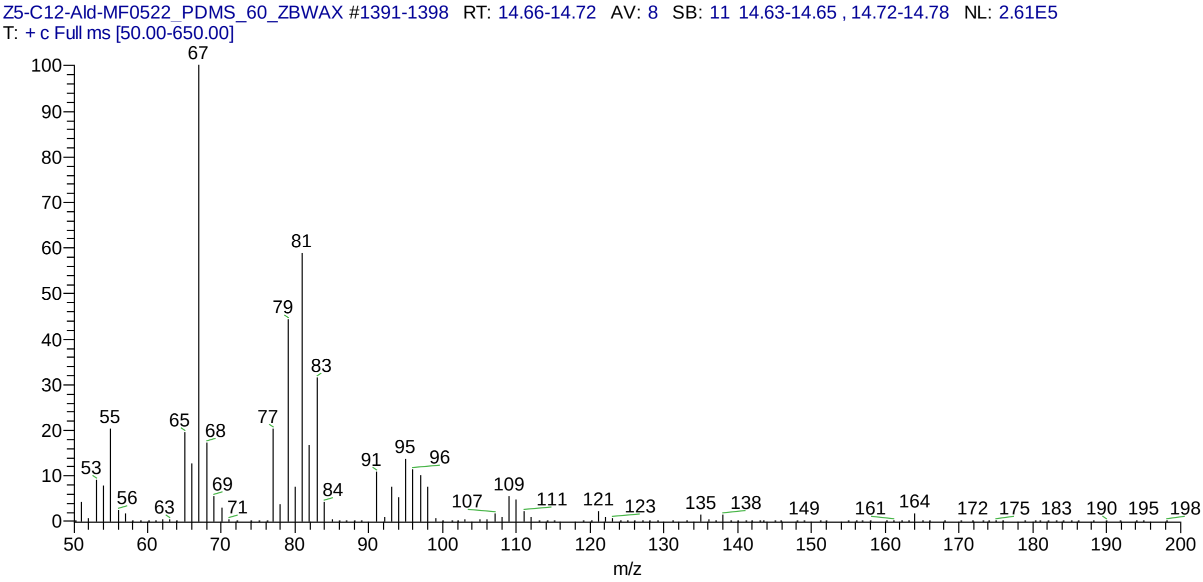

(Z5)-12:Ald

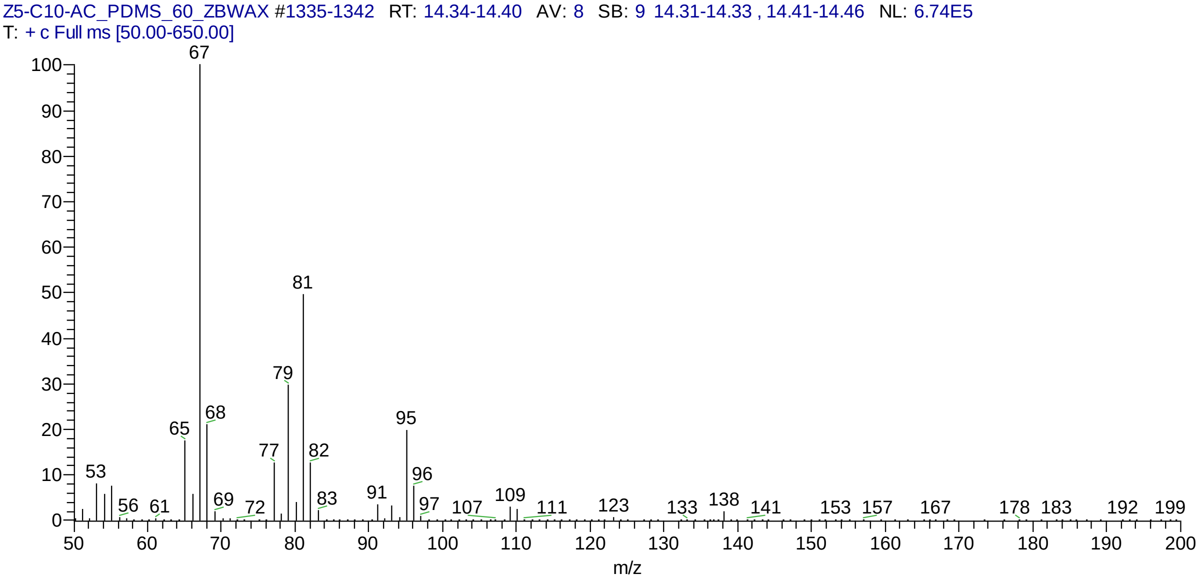

(Z5)-10:OAc

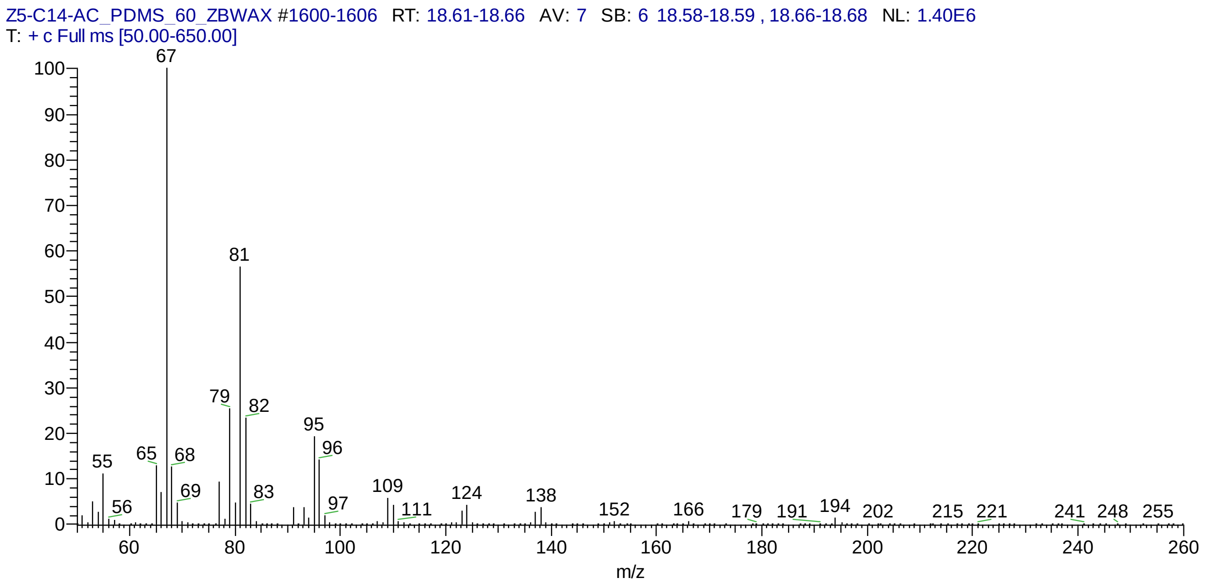

(*Z*5)-14:OAc

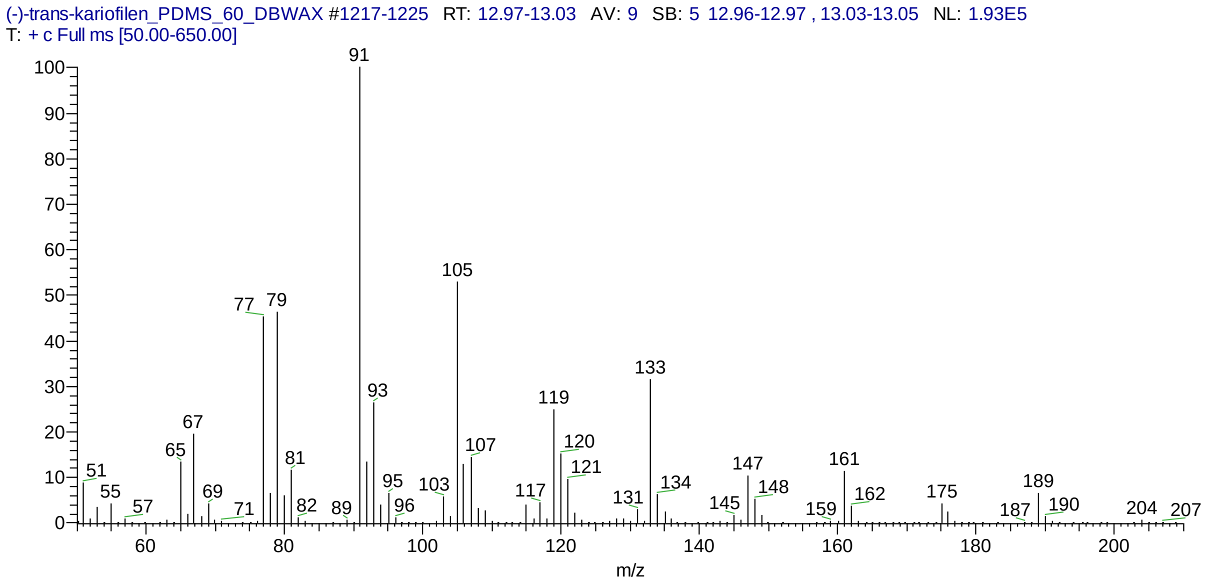

β-caryophyllene

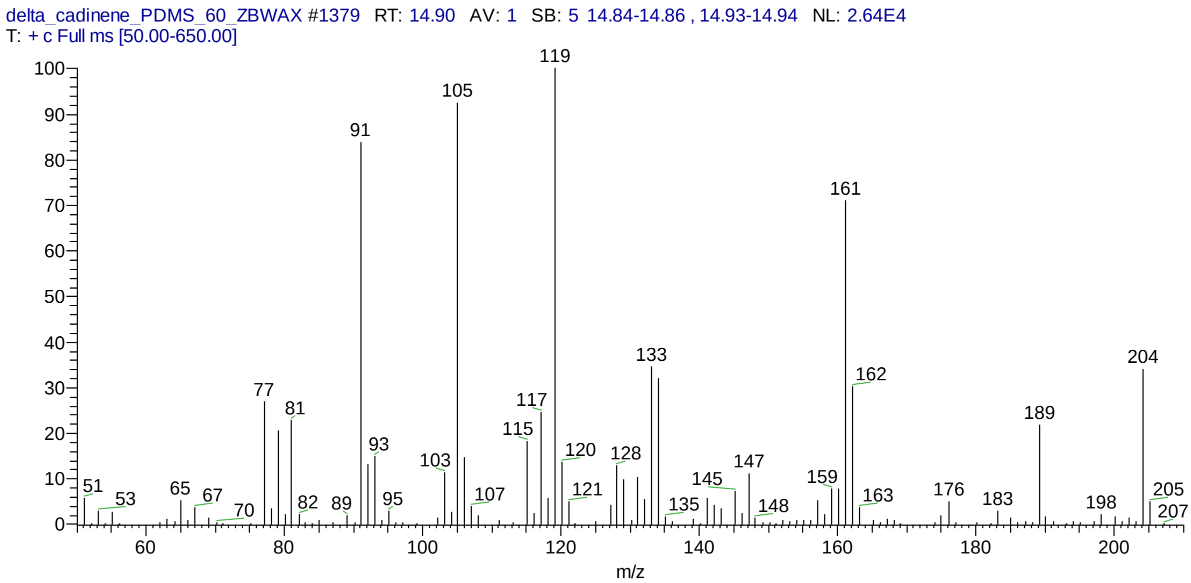

δ-cadinene

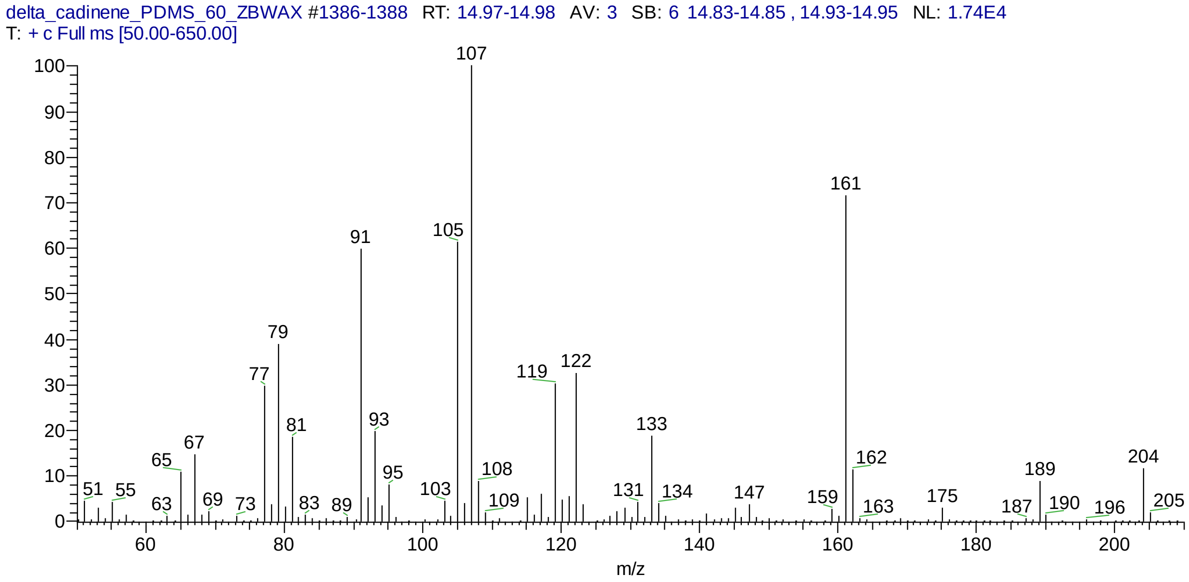

γ-cadinene

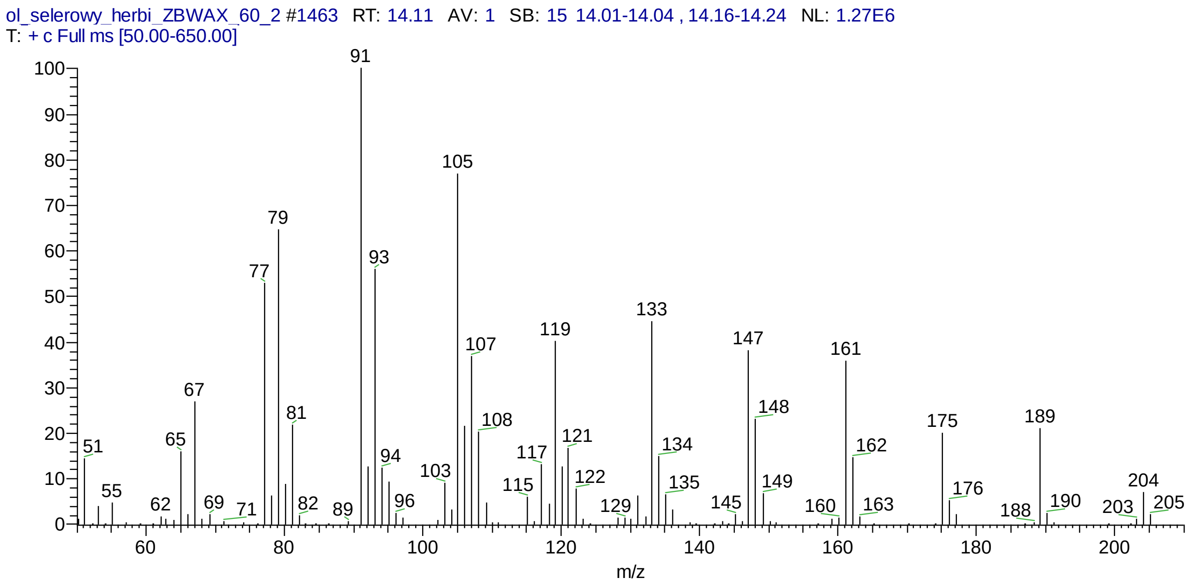

β-selinene

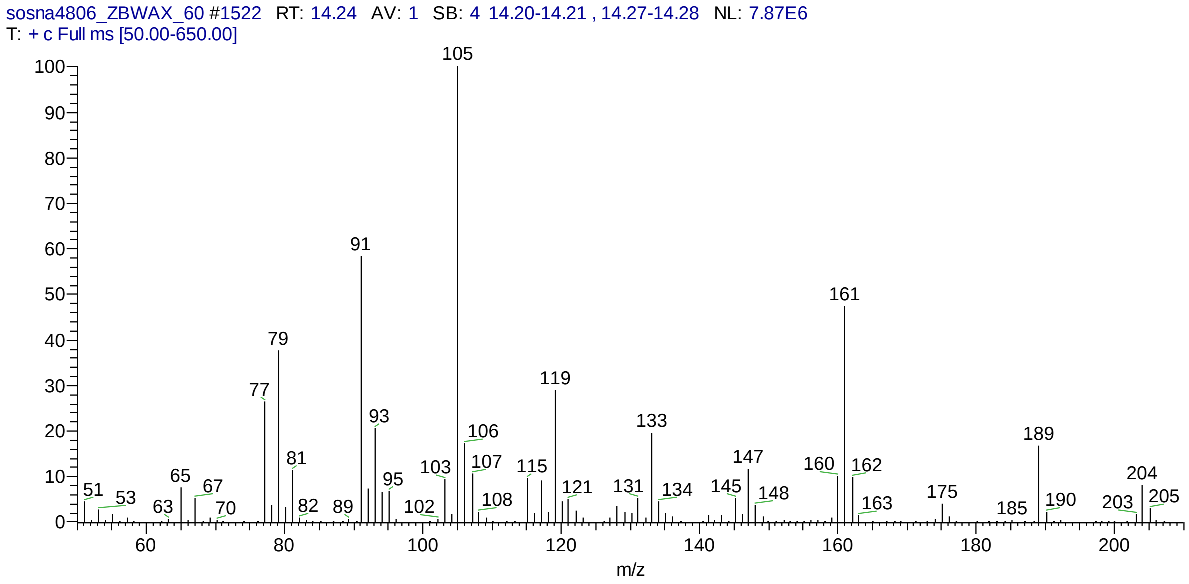

α-muurolene

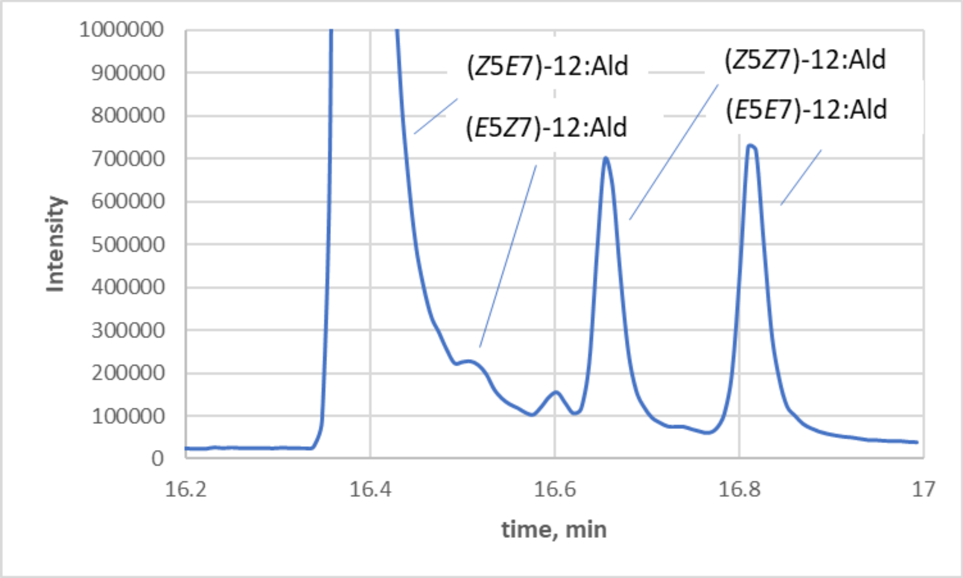
Intensity
time, min
(Z5E7)-12:Ald
(E5Z7)-12:Ald
(Z5Z7)-12:Ald
(E5E7)-12:Ald
(a)

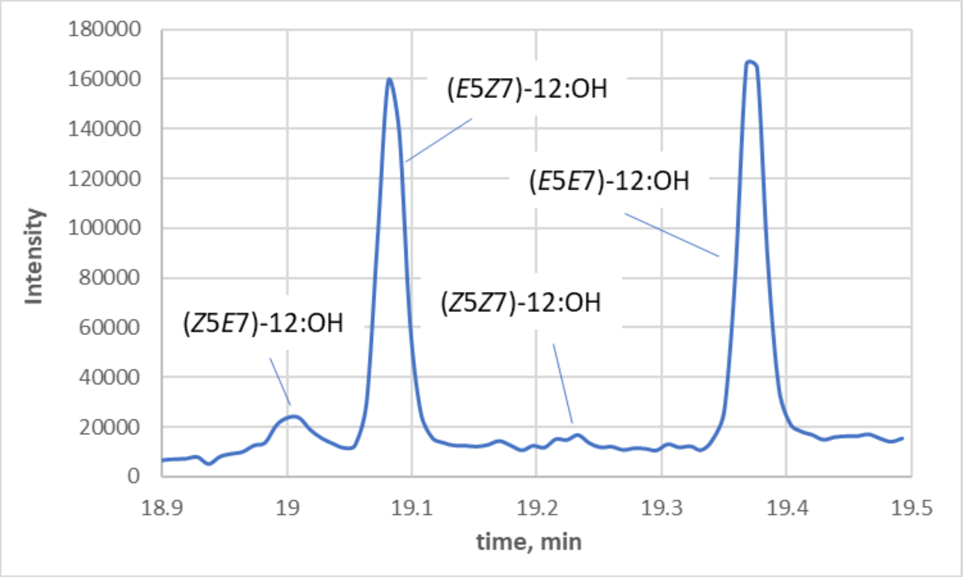
Intensity
time, min
(E5Z7)-12:OH
(E5E7)-12:OH
(Z5Z7)-12:OH
(Z5E7)-12:OH
(b)

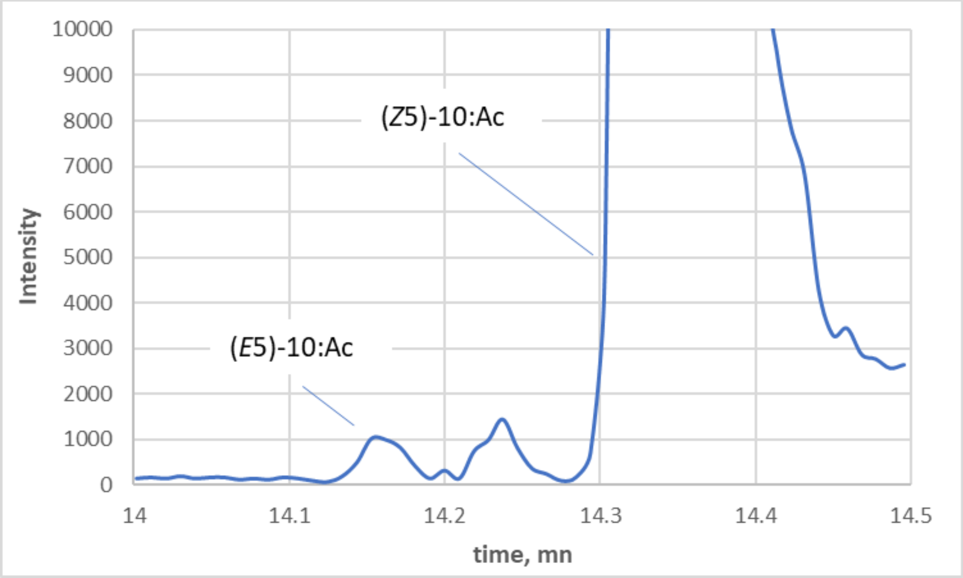
Intensity
time, mn
(Z5)-10:Ac
(E5)-10:Ac
(c)

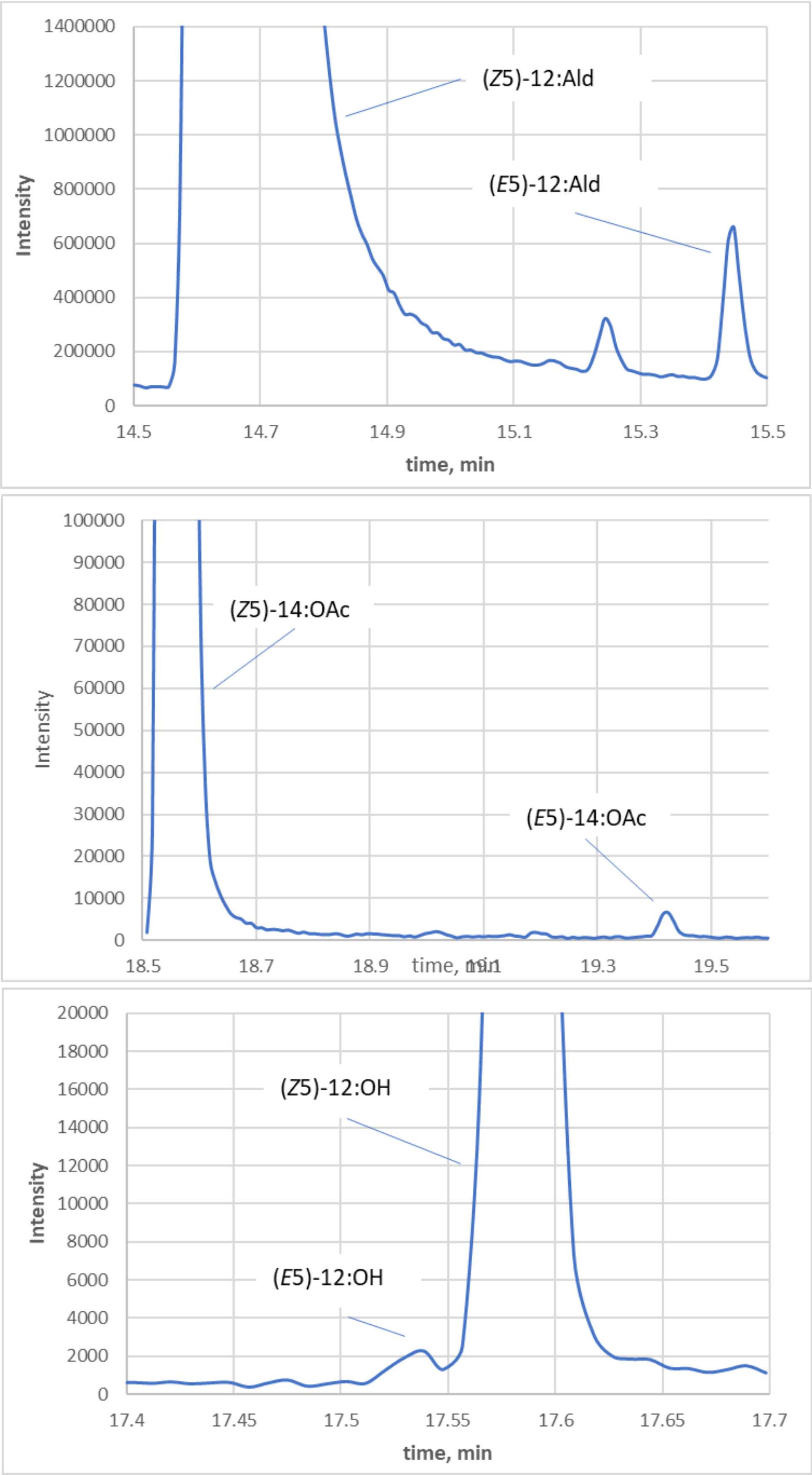

(d)

(e)

(f)

Figure S5. Chromatographic separation of isomers of compounds that were detected in emissions of calling *D. pini* females, obtained using a ZBWAX column. Plates (a) and (b) are Total Ion Chromatograms, plates (c) – (f) are Extracted Ion Chromatograms. Diene separation is consistent with ref. [46] in the main text and monoene separation – with ref. [1, 2] here.

NMR SPECTRA OF LIKELY COMPONENTS OF *D. pini* SEX PHEROMONE IN THIS WORK

1. **(Z5,E7)-5,7-dodecadien-1-ol (Z5,E7-12:OH)** ^{1}H NMR (500 MHz, CDCl3): 6.25 ddd (J=1.0 Hz, 10.5, 14.7 Hz, 1H), 6.00 t (J=10,5, 1H), 5.69 (dt, J=7.5 Hz, 14.7, 1H), 5.25 dt (J=7.5 Hz, 10.5, 1H), 3.65 (t, J=6.0 Hz, 2H), 2.20 q (J=7.5 Hz, 2H), q 2.08 (J=6.8 Hz, 2H), 1.55-1.65 m (2H), 1.43-1.50 (m, 2H), 1.33-1.40 (m, 5H), 0.90 (t, J=7.5 Hz, 3H).
2. **(Z5,E7)-5,7-dodecadienal (Z5,E7-12:Ald)** ^{1}H NMR (500 MHz, $CDCl_3$): 9,81 s, CHO, 6.24 ddd (J=1.4 Hz, 10.5, 14.4 Hz, 1H), 6.05 t (J=10,4, 1H), 5.71 (dt, J=7.4 Hz, 14.7, 1H), 5.32 dt (J=7.4 Hz, 10.5, 1H), 2.55 t (J=6.3 Hz, 2H), 2.92 q (J=7.3 Hz, 2H), q 2.08 (J=6.8 Hz, 2H), 1.55-1.65 m (2H), 1.43-1.50 m (2H), 1.33-1.40 m (5H), 0.92 t (J=7.3 Hz, 3H).
3. **(Z5)-5-dodecen-1-al (Z5-12:Ald)** ^{1}H NMR (500 MHz, CDCl3): 9,78 s (1H); 6,25 dd (J=10.5 Hz, 14.7) 1H; 6,00 t (J=10.5 Hz) 1H; 5,68 dt (J=7.5 Hz, 10.5) 1H; 5,25 dt (J=7,5 Hz, 10,5) 1H;2,43-2,47 m 2H; 2,22 q (J=7,5 Hz) 2H; 2,10 q (J=6,8 Hz) 2H; 1,70-1,80 m 2H; 1,26-1,45 m, 4H; 0,90 t (J=7,5 Hz) 3H.
4. **(Z5)-5-dodecen-1-ol (Z5-12:OH)** ^{1}H NMR (500 MHz, CDCl3): 5,30-5,40 m, 2H; 3,65 t (J=6.5 Hz) 2H; 2,08 dt (J=6.5 Hz, 6.5) 2H; 2,01 dt (J=6.5 Hz, 6.5) 2H; 1,54-1,60 m, 3H; 1,40-1,45 m, 2H); 1,24-1,38 m, 8H; 0,89 t (J=6.8 Hz) 3H.
5. **Z5-decen-1-yl acetate (Z5-10:OAc)** ^{1}H NMR (500 MHz, CDCl3): 5.33 dt (2H, J1=6 Hz, J2=2.1 Hz, CH=CH); 4.03 t (2H, J=6.6 Hz, CH20Ac); 2.00 m (7H, CH2CH=CHCH2, CH3COO); 1.3 m (2H, (CH2)2); 1.3 m (3H, CH3).
6. **Z5-tetradecen-1-yl acetate (Z5-14:OAc)** ^{1}H NMR (500 MHz, CDCl3): 0.87 t (H14, J=6 Hz, -CH3,3H); 1.26 s (H9,H10,H11,H12, -(CH2)4-,8H), 1.37-1.43 m (H3,H8,H13, -(CH2)3-,-6H), 1.60-1.66 m (H2, -(CH2)-,2H), 1.98-2.05 t (H7,H10, -(CH2)2-,4H), 4.06 t (H1, J=6,5 Hz, -CH2- O-, 2H), 5.33 (H5, H6, J=10 Hz,-CH=CH-,2H), 2.05 s (H1, -OCOCH3, 3H)

LIST OF COMPOUNDS IDENTIFIED IN SCOTS PINE ESSENTIAL OIL OBTAINED BY STEAM DISTILLATION (*SPEO*)

(dominating components, as judged by the peak areas, are printed in bold):

**α-pinene**	**3-carene**	**germacrene D**
camphene	limonene	β-selinene
β-pinene	terpinolene	α-muurolene
myrcene	**β-caryophyllene**	**δ-cadinene**

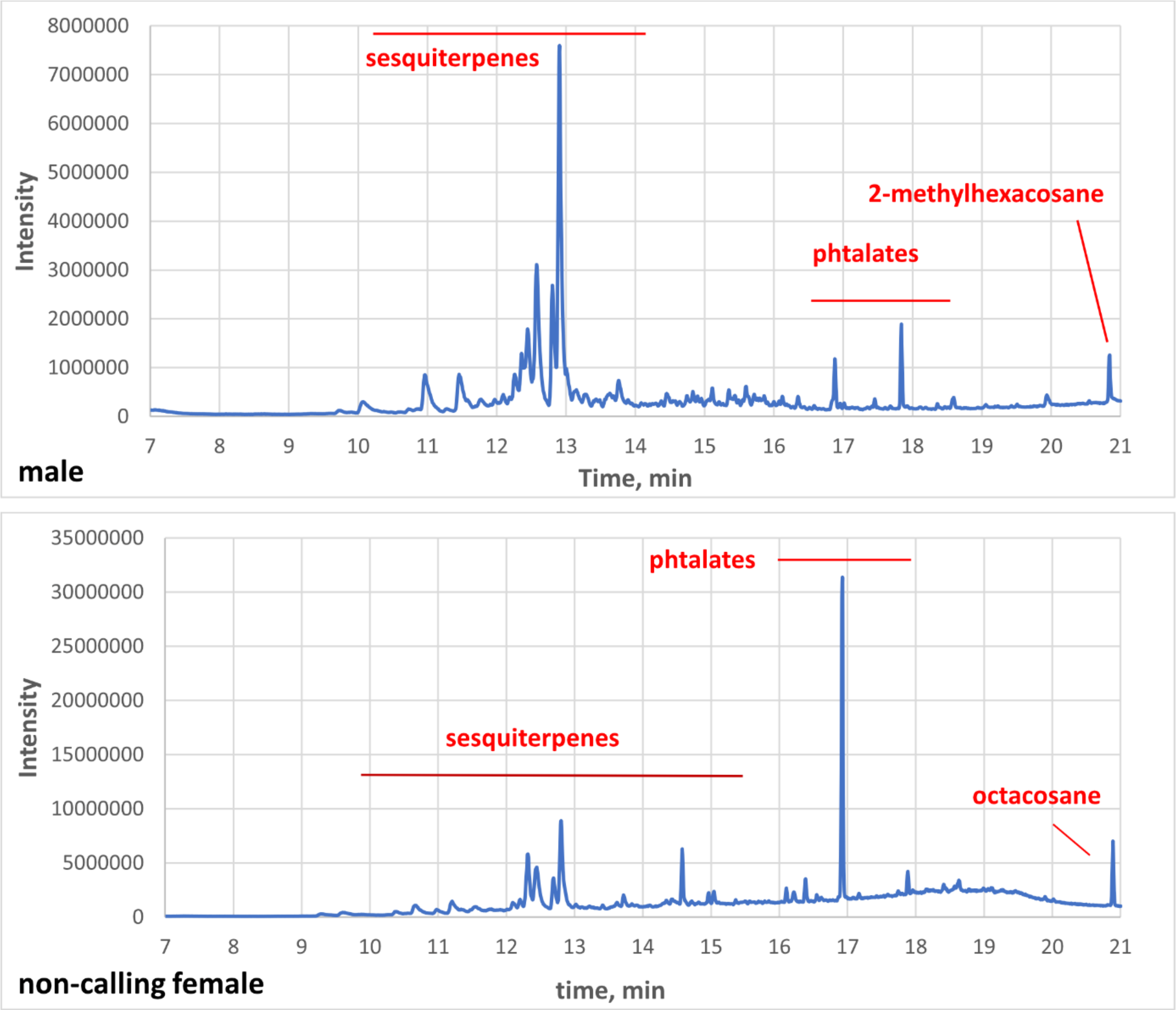

Figure S6. Preliminary chromatograms of SPME samples from *D. pini* moths: male (top panel), and non-calling female (bottom panel), obtained using an Rxi-5ms column. No likely sex-pheromone components were observed.

Table S1. Tunnel experiments without SPEO – number of experiments in which given reactions occurred and effectiveness factors *f* with standard errors

**Reaction**		**Lures**						
nr	weight	MD12	MD14	MD15	MD16	MD17	MD18	air flow
1	0							
2	1							
3	4	7	2	4	3	1	3	15
4	9	3	2		3	3	2	
5	16	1	1	1	1	1		
6	25	1		1	1	1		
7	36		1		2		1	
Total number of experiments		12	6	6	10	6	6	15
f		8.0	13.0	9.5	15.2	12.0	11.0	4
f standard error		1.9	4.9	3.7	4.0	3.0	5.1	

Table S2. Tunnel experiments with SPEO – number of experiments in which given reactions occurred and effectiveness factors *f* with standard errors

**Reaction**		**Lure**					
nr	weight	MD12	MD14	MD15	MD16	MD17	MD18
1	0						
2	1	2					
3	4	8	3	1	2	2	3
4	9	4	1	4	1	1	1
5	16	5	1		2	1	
6	25	4					
7	36	4				2	
Total number of experiments		27	5	5	5	6	4
f		14.59	7.40	8.00	9.80	17.50	5.25
f standard error		2.29	2.36	1.00	2.69	6.12	1.25

REFERENCES

1. Marques, F.d.A.; McElfresh, J.S.; Millar, J.G. Kováts retention indexes of monounsaturated C12, C14, and C16 alcohols, acetates and aldehydes commonly found in lepidopteran pheromone blends. Journal of the Brazilian Chemical Society 2000, 11, 592-599, doi:https://doi.org/10.1590/S0103-50532000000600007
2. Levi-Zada, A.; Fefer, D.; Anshelevitch, L.; Litovsky, A.; Bengtsson, M.; Gindin, G.; Soroker, V. Identification of the sex pheromone of the lesser date moth, Batrachedra amydraula, using sequential SPME auto-sampling. *Tetrahedron Letters* **2011**, *52*, 4550-4553, doi:https://doi.org/10.1016/j.tetlet.2011.06.091.
